# Alterations in the Components of the GABA–Glutamate System During ZIKV Infection: A Neuroscience Approach

**DOI:** 10.3390/ijms27114833

**Published:** 2026-05-27

**Authors:** Aura Caterine Rengifo, Julián Naizaque, Gerardo Santamaría, Diego Alejandro Alvarez-Díaz, Andrea Viviana Rodriguez, Gina Guío-Vega, Jorge Rivera, Carlos Eduardo Castro, Zulma Dueñas, Orlando Torres-Fernández

**Affiliations:** 1 Grupo de Morfología Celular, Dirección de Investigación en Salud Pública, Instituto Nacional de Salud (INS), Avenue 26 No. 51-20–Zone 6 CAN, Bogotá 111321, Colombia; jrnaizaqueg@unal.edu.co (J.N.); gsantamaria@ins.gov.co (G.S.); a.viviana.archila@gmail.com (A.V.R.); jriverari7@gmail.com (J.R.); carlos123castro@gmail.com (C.E.C.); 2 Grupo Investigación y Desarrollo de Vacunas y Biológicos Estratégicos en Salud Pública, Instituto Nacional de Salud (INS), Avenue 26 No. 51-20–Zone 6 CAN, Bogotá 111321, Colombia; dalvarezd@ins.gov.co; 3 Grupo de Neurobiología, Fisiología y Comportamiento, Facultad de Medicina, Universidad Nacional de Colombia, Avenue 30, No. 45-03, Bogotá 111321, Colombia; zjduenasg@unal.edu.co

**Keywords:** NMDA receptors, GABA-A receptors, Zika virus, cerebral cortex, cerebellum, neurotransmitter metabolism and transport

## Abstract

Zika virus (ZIKV) infection is associated with severe neurodevelopmental disorders. However, the molecular mechanisms involved in the imbalance of excitatory and inhibitory neurotransmitter systems remain poorly understood. In this study, we evaluated the expression of components of the GABA–glutamate system in neonatal BALB/c mice inoculated with ZIKV at 10 days post-infection (dpi). Analysis of GABA-A and NMDA receptors revealed widespread downregulation of GABA-A and NMDA receptor subunits in the cerebral cortex and cerebellum, except for the alpha-5 and epsilon GABA-A subunits, which were upregulated in the cerebellum. Infected mice also showed increased GABA immunoreactivity and glutamate loss. The enzymes involved in neurotransmitter synthesis or transport confirmed these findings when assessed by qRT-PCR or Western blot, revealing increased expression of GAD-65/67, accompanied by a loss of glutamate dehydrogenase (GLUD) in the cerebral cortex and cerebellum, along with decreased expression of the glutamate transporter (VGLUT) in the cerebral cortex. These findings suggest that GABA synthesis increases during ZIKV infection, creating a neurochemical environment that favors viral replication and contributes to the migration and synaptic defects observed in congenital Zika syndrome.

## 1. Introduction

Zika virus (ZIKV) is a mosquito-borne flavivirus that has emerged as a global public health concern because of its neurological complications, particularly after the 2015–2016 epidemic [1,2]. Although the virus typically causes asymptomatic or mild disease, ZIKV infection has been conclusively linked to Zika congenital syndrome (CZS) in infants and Guillain–Barré syndrome (GBS) in adults [3,4,5,6]. Many studies have investigated the possible mechanisms of viral action; however, the way in which the virus infects neurons and alters several components of the nervous system is still unknown [7,8,9,10]. 

Among the neurological manifestations of ZIKV infection reported during fetal and early postnatal development in humans and rodents are the presence of calcifications, a decrease in the thickness of the cortical layers, cerebellar hypoplasia, gliosis in the periventricular white matter, ependymal damage, and ocular alterations; malformations that are part of the so-called congenital ZIKV syndrome [6,11,12,13].

In adults, encephalitis, meningoencephalitis, myelitis, and variants of Guillain–Barré syndrome, such as motor axonal neuropathy and inflammatory demyelinating polyneuropathy, have been reported [14]. However, viral tropism toward the central nervous system (CNS) in adults has been reported in only a few studies in elderly patients who are immunocompromised or have some type of comorbidity, and the presence of the virus is not necessarily associated with fatal cases [15]. Despite the emergence of scientific evidence on the neurological manifestations associated with ZIKV infection, whether the pathophysiology of the lesions caused by ZIKV is directly related to the virus or to the toxins released by inflammatory reactions remains unknown [16,17,18,19,20].

Different receptors and cell types have been suggested as targets for the spread of the ZIKV in the central nervous system [21,22]. Within this group are the phosphatidylserine receptors TIM (T-cell immunoglobulin and mucin domain) and TAM (Tyro3, Axl, and Mer), given their role in viral dissemination, as have been demonstrated with other flaviviruses such as dengue virus and West Nile virus [11,23]. AXL receptors are expressed in testicular cells and neuronal progenitor cells, and their mRNA has also been identified in radial glial cells, astrocytes, and microglia [11,23]. Due to its association with infection by various flaviviruses, this receptor was considered a putative target for ZIKV. To test this hypothesis, several studies have infected various cell lines with ZIKV prior to AXL ablation or silencing. Cell lines such as CHME3 (human microglial cells), human astrocytes, and murine astrocytes were able to eliminate susceptibility to flavivirus infection following AXL dysfunction. However, this effect was not observed in hNPCs (neural progenitor cells) or in brain organoids derived from the same lineage [11,24]. These findings have highlighted the need to identify new receptors and cell populations to explain viral entry and damage in non-glial cells, such as CNS neurons.

Previous studies on the pathogenesis of ZIKV have shown changes in the expression of genes involved in neurodevelopment and decreases in the mRNA levels of genes associated with the synthesis of neurotransmitters [25,26,27] and calcium-binding proteins, such as calbindin and parvalbumin [27]. These proteins are present in GABAergic cells, a cell type with a high predisposition to the tropism of viruses that invade the nervous system [28,29,30,31,32,33,34].

Possible effects on the GABAergic system have been reported in models of mosquito infection with ZIKV following gabazine administration, a GABA-A receptor antagonist [35]. In this case, the receptor became hypofunctional after infection, as the drug did not affect excitatory potentials in the infected group, suggesting that the receptor was inhibited by the viral infection. Alterations in GABA-A receptor function have also been demonstrated indirectly in neonatal mice infected with ZIKV at a non-lethal dose and evaluated in adulthood. Souza et al. [36] observed that these mice showed a greater predisposition to seizures after pentylenetetrazol administration. This evidence, along with reports of calcifications in rodent and human brains, may indicate involvement of the GABA–glutamate system during viral pathogenesis. This is due to the importance of glutamate as a precursor of GABA and its role in autophagy [37,38], a type of cell death that ZIKV exploits as a mechanism of viral infection [39].

Regarding the glutamatergic system, certain NMDA receptor antagonists have been shown to inhibit viral replication in neurons infected with the rabies virus [40,41]. In ZIKV infection models, some of these antagonists have been reported to increase neuronal survival but have no effect on viral replication or on the survival of mice infected with the virus [42]. These findings are significant given the growing body of literature proposing a glutamate-associated excitotoxicity pathway to explain the neurological effects commonly reported during infection with neurotropic viruses [42].

GABAergic and glutamatergic systems are involved in various physiological processes, including cellular respiration, reflexes, neurodevelopment, nociception, and neuronal plasticity [43,44,45]; therefore, changes in these systems can severely impair neuronal function [46]. For this reason, various studies indicate that, under pathological conditions, the glutamine–glutamate–GABA amino acid cycle is affected, as are some of its receptors, including the GABA-A receptor and the NMDA receptor. Changes in these systems have been widely linked to behavioral changes and mechanisms of neuronal degeneration [47,48,49].

The function of GABA-A and NMDA receptors and their neurotransmitters can be altered by changes in amino acid transamination cycles, visceral metabolic dysfunctions, or infectious agents that affect them directly or indirectly, as might occur during ZIKV infection [48,50,51,52]. To test this hypothesis, the expression of components of the GABA–glutamate receptor system, neurotransmitters and enzymes involved in their synthesis was evaluated in neonatal BALB/c mice infected with ZIKV [50,51,52].

## 2. Results

### 2.1. Signs of Disease in the Infected Mice

Our research group has previously documented the clinical signs of the ZIKV infection model used in this study [27]. In our model, weight loss became evident 6 days after infection, compared to the mock-infected mice. After 7 days post-infection, infected mice exhibited social isolation, fur bristling, tremors, ataxic gait, decreased activity, tactile hypersensitivity, and hypotonia. The severity of these symptoms increased steadily until the tenth day after infection, when prostration or hind-limb paralysis occurred. At this stage, the animals were euthanized to alleviate suffering and ensure their welfare.

### 2.2. Differential Expression of GABA-A and NMDA Receptors

**Figure 1:**
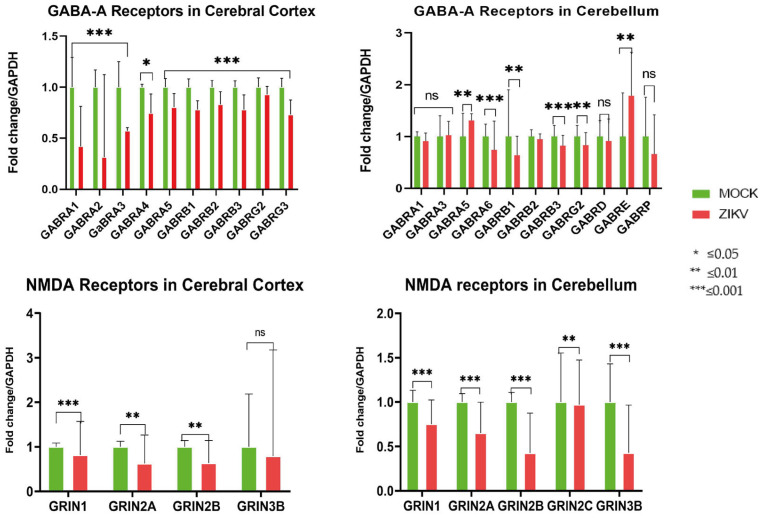
Fold changes in the expression of GABA-A and NMDA receptors in the cerebral cortex and cerebellum of control and ZIKV-infected mice. The mRNAs of the analyzed subunits are expressed until the second postnatal week, and in some cases, their expression varies between the cortex and the cerebellum. These RNAs were downregulated for GABA-A and NMDA in the cortex and cerebellum, except in the cerebellum for GABRA-5, which codes for α5, and GABRE, which codes for ε. The data were analyzed according to the Livak and Schmittgen method [53], and the primer sequences used are described in Table 3 and Table 4. The data represent the analysis of four biological replicates and three technical replicates. Reaction efficiencies and fold changes are detailed in Supplementary Materials Tables S1.1–S1.4. The error bars correspond to the SD. Bar charts created in GraphPad Prism 10.6.1 software and image mosaic created in Adobe Illustrator 1.0.

Subunits expressed up to the second postnatal week were evaluated based on the Allen Brain Atlas (Table 1 and Table 2), given that mice were ZIKV-inoculated on day 1 and euthanized at 10 dpi. The expression of the GABA-A and NMDA receptor subunits was downregulated in the cortex and cerebellum of infected mice, except for the expression of the α-5 and ε subunits of GABA-A in the cerebellum, in which mRNA expression was upregulated (Figure 1).

### 2.3. Molecular Components of GABA and Glutamate Metabolism and Transport

**Figure 2:**
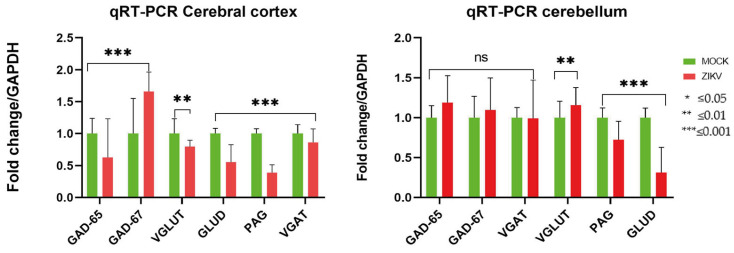
mRNA expression levels of GABA and Glutamate metabolic enzymes and transporters in the cerebral cortex and cerebellum of ZIKV-infected mice. The expression levels of these transcripts varied across brain areas; for example, GAD-65 expression decreased in the cortex but did not change in the cerebellum. The assays showed overexpression of GAD-67 in the cerebral cortex, whereas no significant changes were observed in the cerebellum. VGAT showed loss in the cortex, as did VGLUT, but the latter showed an increase in the cerebellum. The mRNA encoding glutamate dehydrogenase (GLUD) and PAG were downregulated. GAD-65 (glutamate decarboxylase present in the synaptic bud), GAD-67 (glutamate decarboxylase present in the cytoplasm), PAG (phosphate-activated glutaminase), GLUD (glutamate dehydrogenase), VGAT (vesicular GABA transporter), and VGLUT (vesicular glutamate transporter). The data were analyzed according to the method of Livak and Schmittgen [53], and the primer sequences used are listed in Table 5. The data represent the analysis of four biological replicates and three technical replicates. Reaction efficiencies and fold changes are detailed in Supplementary Materials Tables S2.1 and S2.2. The error bars correspond to the SD. Bar charts created in GraphPad Prism 10.6.1 software and image mosaic created in Adobe Illustrator 1.0.

To evaluate some enzymes involved in the synthesis and transport of GABA and glutamate—molecules known as natural ligands of GABA-A and NMDA receptors—qRT-PCR and Western blot assays were performed. The qRT-PCR assays revealed overexpression of glutamate decarboxylase (GAD-67), an enzyme that decarboxylates glutamate for its conversion to GABA, specifically within the cerebral cortex, whereas no significant changes were observed for this enzyme in the cerebellum. Additionally, there was a significant downregulation of the vesicular transporters for GABA (VGAT) and glutamate (VGLUT) in the cortex, as well as a decrease in the levels of glutamate dehydrogenase (GLUD) and phosphate-activated glutaminase (PAG) across the evaluated areas (Figure 2).

**Figure 3:**
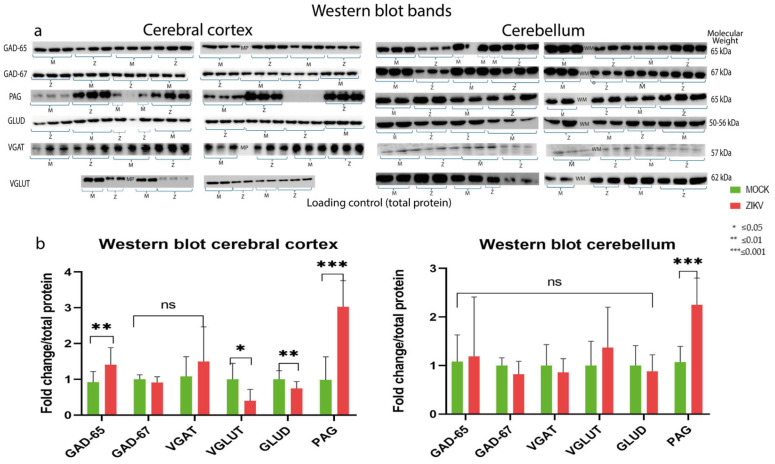
Protein expression levels of GABA and Glutamate metabolic enzymes and transporters in the cerebral cortex and cerebellum of ZIKV-infected mice. (**a**) Protein bands in Western blot assays. (**b**) Quantification of Western blot bands presented in Figure (**a**). Here, an increase in GAD-65 was observed in the cortex and cerebellum without significant changes in the latter, and GAD-67 did not change, but the loss of GLUD and VGLUT in the cortex observed in the qRT–PCR assays was maintained. In all tests, four biological replicates and two or three technical replicates were analyzed. Total protein was used as a normalization control (No-Stain TM Protein Labeling Reagent Kit). Images of membranes with total protein are visualized in Supplement S3. The analysis was performed as previously described [54]. Note the increase in PAG in both brain areas, contrary to that obtained during the q-RT—PCR assays. Fold changes are detailed in Supplementary Materials Tables S4.1 and S4.2. The error bars correspond to the SD. M = Mock; Z = ZIKV; WM = Weight marker. Bar charts created in GraphPad Prism 10.6.1 software and image mosaic created in Adobe Illustrator 1.0.

The results of the Western blot analyses indicated that the behavior of the group of infected mice slightly differed from that observed by the qRT–PCR assays; here, an increase in GAD-65 and a downward trend in GAD-67 were observed in the cortex and cerebellum. An increase in PAG was also detected in both the cortex and the cerebellum, but VGLUT tended to decrease in the cortex but increase in the cerebellum; however, the decreasing trend of GLUD was remarkable in both areas (Figure 2 and Figure 3).

### 2.4. Evaluation of GABA and Glutamate Neurotransmitter Levels

**Figure 4:**
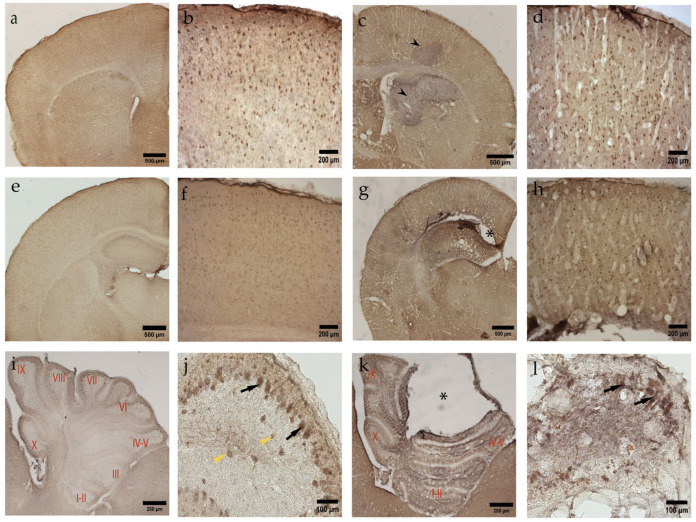
Evaluation of GABA expression in the brain tissue of BALB/c mice that were mock-infected with ZIKV at 10 dpi. Panoramic images and enlargement of coronal sections of the brain (**a**,**b**). Anterior cerebral or motor cortex of a mouse panoramic control (**a**) and enlargement of the cortical (**b**) and infected (**c**,**d**) layers. Note that in the infected tissue, the milky aspect (arrowhead) corresponds to areas with calcifications and the greatest number of GABA-positive neurons compared with the control. Posterior or somatosensory cerebral cortex of control mice and enlargement of the cortical layers (**e**,**f**) and infected (**g**,**h**). Panoramic view of a sagittal section of the cerebellum of a control mouse (**i**) and enlargement of folia IX (**j**) and an infected mouse ((**k**,**l**) showing the delimited cytoarchitecture of the Purkinje cell layer (arrow) and a few neurons of the Golgi in the granular layer (yellow arrowhead). In the cerebellum of infected mice (**k**), tissue damage is observed because of the formation of calcifications, with a greater tendency toward folia VII and VIII (asterisk). In the enlarged image (**l**), a disordered and incomplete Purkinje layer is observed, making visualization of Golgi neurons difficult. Technique: Free-floating immunohistochemistry for GABA using the DAB–Nickel system. Image mosaics created in Adobe Illustrator 1.0.

When GABA immunodetection assays were performed in mock-infected and infected mice, an increase in GABA immunoreactivity was detected, with a total of 285 ± 59 GABA+ cells in mock-infected mice vs. 413 ± 33 in infected mice (*p* value = 0.03) in the anterior or motor cerebral cortex. For the most posterior or somatosensory cortex, values of 256 ± 56 GABA+ cells were found in the mock-group vs. 395 ± 14 in the infected group (*p* value = 0.0079) (Figure 4, Tables S5.1 and S5.2 in Supplement S5).

**Figure 5:**
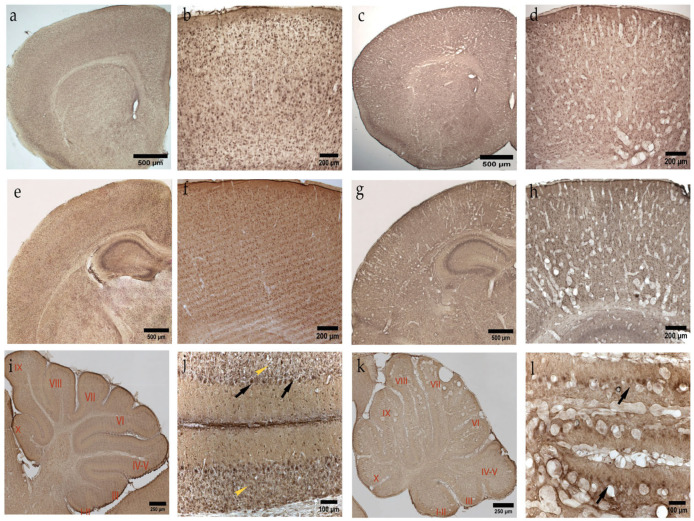
Evaluation of glutamate expression in the brain tissue of BALB/c mice at 10 dpi after ZIKV infection. Panoramic images and enlargement of coronal sections of the mock (**a**,**b**) and infected (**c**,**d**) cerebral cortex. Posterior or somatosensory cerebral cortex of control mice in panoramic image and magnification of cortical layers in control (**e**,**f**) and infected (**g**,**h**) mice. In the control tissue, a dense number of neurons distributed throughout all cortical layers was observed; in the infected tissue, the fewest glutamate+ neurons, accompanied by extravasations, were observed. Cerebellum of control mice (**i**) and enlargement of folia IV and V (**j**), and infected mice (**k**,**l**). In the cerebellar tissue, dense marking is evident in the granular layer of all the folia of the cerebellum (yellow arrowhead) and some Purkinje cell nuclei (arrow). In the infected cerebellum, diffuse staining is observed, with less immunoreactivity in the granular layer. Free-floating immunohistochemistry for glutamate by the DAB–Nickel system. Bars (**a**,**b**), 560 μm; (**c**,**d**), 450 μm; inserts 225 μm. Image mosaics created in Adobe Illustrator 1.0.

Glutamate immunodetection assays revealed results contrary to those of GABA; a decrease in the number of neurons positive for the neurotransmitter in the motor cortex was found, with values of 1733 ± 226 in the mock group vs. 1289 ± 234, *p* value = 0.03, in the infected group. In the somatosensory cortex, the values were 1547 ± 161 in the mock group and 1099 ± 50 in the infected group (*p* value = 0.0079) (Figure 5, Tables S5.3 and S5.4, in Supplement S5).

Analysis of the cerebellum revealed a decrease in the number of neurons in the Purkinje cell layer, with a total of 600 ± 9 cells in all layers in the control group and 275 ± 6 in the infected group. This finding contrasts with the optical density analysis, in which an increase in GABA immunoreactivity was detected in the neurons of this layer in the infected group relative to the mock group (Figure 4j,l, Tables S6 (1–4) in Supplement S6).

Immunoreactivity for glutamate in the cerebellum tended to be like that in the cortex; that is, loss of the neurotransmitter was detected. Optical density measurements revealed a greater amount of transmitted light in the infected group, indicating a decrease in the granular layer for the infected group (Figure 5j,l, Tables S6 (5–6) in Supplement S6).

## 3. Discussion

### 3.1. Changes in GABA Metabolism and Transport

Among the biochemical changes observed, an increase in GABA immunoreactivity was detected in the cortex and cerebellum of mice infected with the ZIKV (Figure 3 and Figure 4). These results suggest enhanced neurotransmitter synthesis, supported by the elevated levels of GAD-67 and GAD-65 mRNA or protein observed in the cerebral cortex. These enzymes are responsible for the conversion of glutamate to GABA at the cytosolic and synaptic levels, respectively. Notably, the increase in GAD-65 levels is highly significant because it confirms that ZIKV infection promotes GABA synthesis at the neurotransmitter release site [55].

Despite the increase in GABA levels, the VGAT level did not tend to increase, as expected; in contrast, the results revealed a decrease in the mRNA in the cerebral cortex. Similar results have been reported for VGAT-*knockout* mice in which the absence of the transporter or the loss of its expression resulted in an increase in GABA and glycine levels in the forebrain of E18.5 *Vgat* ^−/−^ embryos without changes in the expression levels of glutamate. These mice presented cleft palate and omphalocele in addition to changes in body size [56], and these results highlight the importance of VGAT and GABA levels as key morphogens in developmental processes. In *C. elegans* models, inactivation by mutations of the functional orthologous gene of VGAT unc-47 resulted in an increase in the number of GABA-immunoreactive neurons, indicating once again the relationship between the loss of this metabolic intermediate and the increase in neurotransmitter levels [57].

The results illustrated in Figure 2 and Figure 3 might suggest a discrepancy between the mRNA and protein levels of the assessed markers. However, this divergence has been widely documented in biological systems, particularly during viral infections and cellular stress, where the correlation between transcription and protein abundance can be remarkably low [58,59]. In the specific context of the ZIKV, flaviviruses have been shown to uncouple host translation from cellular stress responses, allowing certain protein levels to remain elevated or persist with greater stability even when global host translation is suppressed, and mRNA templates are depleted [59]. Applying this to our model of ZIKV infection and the neurotransmitter systems evaluated, it could be argued that ZIKV uncouples host defense responses to cellular stress and promotes the expression of proteins that induce GABA synthesis, thereby creating an environment that fosters immunosuppression and facilitates viral replication [60,61]. Increased levels of GABA have also been reported in in vivo and in vitro models of infection with Japanese encephalitis virus (JEV). In this study, it was found that during viral infection, an aberrant glutamine–glutamate–GABA cycle occurs that increases the synthesis of glutamine in the early stage of viral infection but decreases it in the final phases, accompanied by an increase in GABA immunostaining and GAD-67 enzyme expression. In addition, the authors reported that the addition of GABA at certain concentrations favored viral replication, whereas the addition of α-ketoglutarate and glutamate inhibited viral replication by promoting the tricarboxylic acid (TCA) cycle [60].

According to our findings and those reported from JEV studies, the increase in GABA could indicate an immunosuppressive effect [60], and the occurrence of several events is associated with failures in proliferation, neuronal migration, and chemical synapses, as well as the facilitation of viral dispersion, because neurotropic viruses have a high affinity for GABAergic cells [28,32,40,62]. The GABAergic system plays a determining role in neuronal synapses because γ-aminobutyric acid (GABA) is the main inhibitory neurotransmitter in the adult mammalian CNS due to its action on ionotropic and metabotropic receptors [52,63,64]. 

In the ventricular zone of the rat embryonic neocortex, the depolarizing action of GABA leads to a decrease in DNA synthesis and in the number of progenitor cells [65]. Therefore, the increase in cortical GABA that we observed could inhibit the proliferation of cortical neurons and the premature induction of cell differentiation. This hypothesis has been supported by observations from several ZIKV infection models. Assays with mouse and human neural stem cells inoculated with the virus have shown that infection increases differentiation into astrocytic progenitor cells and reduces the number of progenitors of neurons and oligodendrocytes [66,67,68].

In previous studies by our group, an increase in GFAP levels in the cortex and cerebellum of mice infected with ZIKV was reported [27]. This effect could be due to the increased activity of astrocytic cells in response to the increase in GABA under pathological conditions. The effect of ZIKV on the proliferation of astrocytes has been demonstrated through transcriptomic studies performed on cultures of stem cells infected with ZIKV.

Some studies have shown that ZIKV downregulates the expression of genes that promote neurogenesis, such as ARTN2, BHLH, SATB2 and the neurogenic differentiation gene NEUROD1 [68]. Moreover, ZIKV increases the expression of astrocyte gliogenesis factors such as GFAP, ALDH1 and calretinin, which are calcium-binding proteins that act as markers of GABAergic cells [67]. ZIKV could promote astrocytic proliferation to enhance viral fitness, as these cells serve as excellent viral reservoirs and are highly resistant to flavivirus infection [61].

In addition to promoting astrocyte activity, ZIKV may generate an increase in GABA as a response to mitigate inflammation; for example, the peripheral mononuclear cells and CD4+ T cells of patients with diabetes inhibit a greater number of cytokines at a concentration of 100 nM GABA compared with cells obtained from healthy participants [69]. Similarly, in mouse models, infection with SARS-CoV-2 and treatment with GABA decreased the mortality rate, pulmonary edema and viral load, in addition to the production of the proinflammatory cytokines TNF-a, IL-6, IP-10 and TLC2 [70].

Despite the possible beneficial effects of GABA, this amino acid or agonists such as diazepam may exacerbate poxvirus infections such as those caused by cowpox and vaccinia virus. Six-week-old BALB/c mice treated with diazepam and inoculated intranasally with one of the viruses showed greater signs of disease, weight loss, and necrotic tissue and a lower level of antibodies against the respective viral agents, indicating an immunosuppressive effect of diazepam or GABA, and a decrease in the number of polymorphonuclear cells has also been reported [71].

### 3.2. GABA-A and ZIKV Receptors

The GABA-A receptor is among the protein complexes to which GABA binds and is among the most studied neuronal receptors because of its high therapeutic potential as a possible neuroprotector. Proper functioning of this receptor protects neurons from neurodegeneration caused by calcium imbalance, a phenomenon known as excitotoxicity; thus, alterations in this activity have been associated with neurodegeneration and behavioral disorders [72,73].

We evaluated the subunits of GABA-A receptors that are expressed until the second postnatal week in mice and that coincide with the receptors expressed in humans between the first and second postnatal years, which is a critical period during which important hanges in synaptogenesis and neuronal plasticity occur [74]. The levels of the evaluated GABA-A receptor subunits were downregulated in the cortex and cerebellum of mice inoculated with ZIKV, except for the subunits α5 and ε, whose expression was upregulated in the cerebellum of infected mice (Figure 1).

Subunit α5 is found in the cytoplasmic domains M3 and M4 associated with isoforms α1-3, β1-3, γ2S or γ2L of the GABA-A receptor and scaffolding proteins radixin and gephyrin. In addition, treatment with GABA results in the phosphorylation of radixin and the recruitment of subunit α5 to the extrasynaptic space [75]. The sequestration of alpha 5 could prevent its protein degradation, as a mechanism that the virus could use to favor cellular phenotypes that facilitate viral replication [61,67,68,76].These data [59,61,67] are of relevance because our results suggested an increase in the synthesis of the neurotransmitter GABA in the cortex and cerebellum (Figure 5) of mice infected with ZIKV accompanied by an increase in the expression of α5 (Figure 1).

The increase in mRNA levels for α5 could also generate dysfunction in the synapses of the human and mouse frontal cortex because the α5 subunit is found in pyramidal neurons and in parvalbumin-positive interneurons of this cerebral area [77]. In other studies, conducted by our working group, dendritic pathology has been found in the pyramidal cells of the cortex and in the Purkinje neurons of the cerebellum of mice infected with the ZIKV (unpublished results). In other studies of ZIKV, we also found increased expression of parvalbumin in the cerebral cortex [27], a calcium-binding protein present in GABAergic interneurons. The increase in parvalbumin increases GABA synthesis, and this could be interpreted as a sign of overstimulation of the GABAergic system when GABA plays an excitatory role, i.e., within the age range evaluated in the present study.

Receptors containing subunit α5 are expressed at high levels during the peak of synapse formation in the second postnatal week in rodents, positioning this subunit as a key protein for modeling the architecture of developing circuits [78]. Changes in expression levels of α5 could lead to two possible scenarios: one in which the total elimination of the gene does not produce changes in the migration or maturation of new neurons and another in which partial inactivation generates serious deficits in neuronal migration and development of dendritic arborizations [79].

Some studies have reported that a reduction in inhibition mediated by subunit α5 of the GABA-A receptor improves functional and neuromorphological deficits in a murine model of Down syndrome (Ts65Dn) [80]. Ts mice treated with allosteric inhibitors of α5 presented improvements in learning and spatial memory, as well as in the dendritic tree, both in the length of the dendrites and in the number of intersections [80]. Cognitive improvements resulting from the inhibition of α5 have also been reported in models of total ablation of the receptor or of forms of the receptor insensitive to benzodiazepines [75].

When comparing the levels of precision required for α5 in relation to our results, we hypothesize that the increase in its mRNA generates two possible scenarios. In the initial stage of infection and when GABA is excitatory, the depolarizing activity necessary to promote neuronal proliferation and migration is attenuated. In later stages, as GABA becomes an inhibitory, excess inhibition mediated by α5 can prematurely halt neuronal maturation by preventing the formation of synapses and their functional integration in emerging circuits, resulting in aberrant wiring and defective synaptic pruning. This theory is supported by studies of α5 on the pyramidal neurons of the hippocampus, where it was reported that the excess inhibition of α5 on NMDA receptors prevents dendritic depolarization, preventing these receptors from reaching the threshold required for their unblocking [78].

Our findings, in conjunction with current knowledge about the behavior of the α5 protein, suggest that the increase in GABA caused by the ZIKV may promote the incorporation of this protein into the synaptic cleft, generating signals that contribute to the malformations observed in ZIKV congenital syndrome. Regarding the role of the ε subunit, which was overexpressed in the cerebellum of ZIKV-infected mice, knowledge of its contribution to proliferation, migration, differentiation, or synaptic establishment remains limited. Upregulation of this subunit has been primarily reported in the lateral cerebellum of patients with psychiatric disorders, including schizophrenia, bipolar disorder, and major depression [81]. The increase in ε could lead to aberrant GABAergic transmission within the cerebellum and its connections to other regions, such as the prefrontal cortex—an area severely affected during ZIKV infection.

In addition, the downregulation of the other GABA-A subunits evaluated in our study could also indicate a dysfunction in receptor response. After primary cultures of Aedes aegypti mosquito neurons were inoculated with ZIKV, Gaburro et al. [35] reported an increase in electrical activity in infected cultures compared with the control. In this study, an increase in electrical response was also observed in control cultures treated with gabazine, a noncompetitive antagonist of GABA-A receptors, but no significant changes were observed in infected cultures [35]. These findings indicate that viral infection can block GABA-A receptor activity, leading to an increase in the virus-promoted excitatory state.

### 3.3. Changes in Glutamate Metabolism and Transport

Dysfunction in glutamate metabolism can alter GABA synthesis since glutamate is its main precursor, and GABAergic cells recruit glutamate generated by glutamatergic neurons and astrocytes for GABA synthesis. For this reason, different studies have indicated that under pathological conditions, the glutamine–glutamate–GABA amino acid cycle and the function of some of its receptors, including GABA-A and NMDA, are affected. Changes in these systems are widely related to alterations in behavior and mechanisms of neuronal degeneration [47,48,81]. The function of these systems can also be altered by changes in the transamination cycles of amino acids, visceral metabolic dysfunctions or infectious agents that affect them directly or indirectly [40,47,48].

The loss of glutamate that we observed is reinforced by the decrease in the expression of the enzyme glutamate dehydrogenase GLUD, which is the transaminase responsible for the interconversion of glutamate to a-ketoglutarate and the vesicular glutamate transporter VGLUT. We detected lower mRNA levels for GLUD in the cortex and cerebellum and for VGLUT in the cortex, with increased levels of VGLUT in the cerebellum. The loss of Phosphate-Activated Glutaminase (PAG), the enzyme responsible for the transformation of glutamine to glutamate, was detected only at the mRNA level, in contrast to the increase in protein levels detected in both brain areas.According to our results, the loss of glutamate immunostaining is strongly linked to reduced GLUD expression and reduced release into the synaptic cleft, at least at the cortical level, due to low VGLUT levels. Glutamate loss has also been reported in other studies of ZIKV infection. Work by Benazzato et al. [82] on neurons derived from dedifferentiated fibroblasts from children with congenital ZIKV syndrome (CZS) showed a decrease in glutamate levels measured between 1 and 96 h post-culture. The authors also found a loss of synapse-associated proteins, such as synapsin 1 and PSD-95. Similar results were also reported by Sow, A. et al. [83] in studies with zebrafish embryos inoculated with ZIKV into the yolk sac two hours after fertilization. Here, a reduction in the number of glutamatergic neurons and a decrease in the glutamate vesicular transporter VGLUT were observed [83].

Our results and the reports by Benazzato and Sow were consistent with those of the studies by Li et al. [60]. on JEV, where a decrease in the synthesis of glutamate and GLUD was reported in the final stages of infection, indicating that the use of glutamate as a guardian of the antiviral response, given that the silencing of GLUD and the a-ketoglutarate dehydrogenase favored viral proliferation.

The increase in PAG could correspond to poor protein degradation during ZIKV infection or to an increase in glutamine levels because of changes in glutamate levels in astrocytes due to the glial role of glutamate [84]. A very likely explanation is that astrocytes are GABAergic cells, and these cells use glutamate as a substrate for the synthesis of GABA and express markers of GABAergic cells, such as GAD-67 and subunits of GABA-A and GABA-B receptors [85].

The increase in PAG could also be due to modifications in the expression of TNF-a because the addition of this cytokine to microglial cultures increased the concentration of glutamate and upregulated the mRNA expression of the PAG enzyme [86]. Similar evidence was found in cultures of microglia infected with Japanese encephalitis virus (JEV), where a pulse with TNF-a increased protein expression according to Western blot assays [87]. An increase in PAG was also reported in a norovirus model because the viral protein NS1/2 increases glutaminase activity and glutaminolysis as a mechanism that favors viral replication [60].

Dysfunction of the glutamatergic system as a mechanism of infection has also been one of the hypotheses concerning the neuropathogenesis of a-pseudorabies herpesvirus (PVR). Mice inoculated subcutaneously showed loss of expression of the NR2A and NR2B subunits of the NMDA receptor; here, treatment with the NMDA agonist improved the survival rate and reduced T-cell cytotoxicity, but the use of the antagonist MK-801 exacerbated the expression of inflammatory cytokines [88].

In contrast to our results and studies on PVR, other investigations have reported findings that seem to indicate an increase in glutamate synthesis, reinforcing the hypothesis of excitotoxicity by the neurotransmitter. Olmo et al. [89], after infecting primary cultures of cortico-striatal neurons with ZIKV, reported increases in glutamate levels by indirect measurements of the fluorescence increase in the production of NADPDH in the presence of glutamate dehydrogenase type II and NADP+ up to 72 hpi. The authors reported no changes in glutaminase levels. The same study reported an increase up to 24 hpi in EAAT, known as SLC1a in mice and as EAAT3 in humans, a sodium-dependent glutamate transporter responsible for eliminating glutamate that did not bind to receptors in the synaptic cleft, which is mainly found in neurons [89].

In the studies of Gaburro et al. [35], mosquito neurons infected with ZIKV showed an increase in mRNA levels of one of the EAATs, GDH1 (also known as GLUD1), and voltage-gated sodium channel (VGNaC) at 72 h post-inoculation (hpi), while no change was detected in VGLUT expression. Additionally, at 24 hpi, GABA vesicular transporter (GAT1) expression increased, and some GABA-A subunits were lost, although the study does not specify which subunit was evaluated [35].

### 3.4. NMDA Receptors and ZIKV

NMDA receptors are the main receptors responsible for neuronal excitation and are among the targets to which glutamate binds. The expression of glutamate receptors varies according to the stage of prenatal or postnatal development [90], and there are equivalencies between humans and rats. The function and expression of the receptors are considered to be equivalent for the first two years of development in humans and the first two weeks of development in rodents [74]. Hyperfunction or hypofunction of NMDA receptors is associated with psychiatric disorders and neurodegenerative disorders [49]. Therefore, NMDA receptors have been the target of study using different approaches.

In this study, we detected a loss of the mRNA of all the subunits of the NMDA receptor in the cortex and cerebellum of mice infected with ZIKV (Figure 1), except for GluN3B, also known as GRIN3B, where, despite the tendency toward loss, no significant changes were observed. Our results differ from those of the studies of Olmo et al. [89] in cortex plus striate cell cultures infected with ZIKV, previously described in the discussion of glutamate. These authors reported an increase in the expression of GLUN2B at 24 h post-infection and a decrease in the rate of cell death after the use of ifenprodil, a noncompetitive antagonist that binds to the N-terminal NTD domain of the GLUN2B subunit. The use of ifenprodil completely blocked the increase in calcium in cells infected by ZIKV, indicating that cell damage could be associated with increases in the activity and expression of NMDA receptors.

Costa et al. reported [42] a decrease in the cell death rate of neurons infected with ZIKV from C56BL/6 corticostriatal cultures beginning on embryonic Day 15 after the use of NMDA antagonists such as memantine, MK-801, agmatine and ifenprodil. These drugs increased the survival rate but had no effect on the viral load. However, in the same study, the survival of adult SV129 *Inf*-α/β R ^−/−^ mice inoculated intravenously with ZIKV did not increase after oral administration of 30 mg/kg memantine [42].

The differences found by us and those reported by several authors could be due to the infection models and the evaluation times; in these other studies, in vitro models were used, and measurements were made up to 48 hpi, while in vivo models were used in our assays, and measurements were performed 10 days post-inoculation when the mice were in the final phase of the disease.

These findings, together with the comments in the discussion section on glutamate metabolism, suggest that the hypothesis of a glutamate-mediated excitotoxic environment proposed by some authors has not been substantiated across the various infection models [91]. Furthermore, the use of NMDA antagonists, such as memantine or ifenprodil, has not reduced viral load or increased survival in infected mice treated with these drugs [42]. This underscores the need to initiate studies with receptor agonists in viral infection models.

### 3.5. Use of Newborn Mice, Signs of the Disease, and Their Correlation with Immunohistochemical Changes

The cerebral cortex in mice originates and develops between embryonic day E12 and postnatal day P8 (E12–P8), but maturation, or so-called arealization, occurs between days E15–P8 [92]. In the present study, the mice were inoculated on day P1 and euthanized at 10 dpi; during this phase, the virus acted on the cerebral cortex during the migration period and at a stage of the synaptogenesis process when neurons are already in their target location, whereas for the cerebellum, the virus affected the phase of migrating neurons, given that their migration culminates on day P21 in mice [65,93] and between the 11th and 20th months of the postnatal period in humans [94,95]. These differences in migration could explain the greater tissue damage in the cerebellum compared to the cerebral cortex, as shown in Figure 4k,l and Figure 5k,l, with notable alterations in the GABA-positive Purkinje cell layer (Figure 4l) and the glutamate-positive external and internal granular layers (Figure 5l).

Newborn mice serve as an ideal model for ZIKV infe68ction due to their immature immune system, which diminishes the interferon I response and facilitates viral degradation of factors such as STAT-2 through alternative pathways different from degradation by NS5 viral protein, such as inhibition of de novo host protein synthesis, which is responsible for antiviral responses [96,97]. Susceptibility to infection decreases with age in mice, particularly when peripheral inoculation occurs after the first week of life [97,98,99], as observed with other flaviviruses, such as dengue [100]. Mice aged 1–3 weeks remain susceptible to ZIKV neuroinvasion because their blood–brain barrier has fewer tight junctions, reduced levels of mature vascular markers, and increased numbers of immature endothelial cells [101]. These mice also display reduced expression of proteins regulating endothelial transcytosis, such as Mfsd2 [102,103]; an interesting aspect because ZIKV uses transcytosis as a mechanism for viral spread [104].

Day 1P of mouse brain neurodevelopment corresponds to nearly half of the human gestational period, approximately week 23 of gestation [105]. This is particularly relevant given that more severe malformations associated with ZIKV congenital syndrome (ZCS) have been reported during the first two trimesters [7,106]. At this stage, many processes occur in parallel, such as neurogenesis in layers II/III and IV of the cerebral cortex, the onset of action potentials in the retina, a considerable increase in the emergence of optic nerve axons, and the expansion of the subventricular zone to promote cortical development [99], during this stage and up to the second postnatal week in mice and the first and second years of life in humans, GABA exerts excitatory functions to give rise to the GABA switch and exert an inhibitory effect.

During early neurodevelopment, ZIKV appears to affect the GABAergic system by interfering with its excitatory functions and, likely, by inhibiting neuronal proliferation, due to an environment that increases susceptibility to infection and neuronal damage, especially when considering the effects of increased GABA on facilitating immunosuppression and its influence on glial phenotypes such as astrocytes, thereby promoting an environment conducive to greater viral replication [61,67,68,76].

Another advantage of neonatal mouse models of ZIKV infection is that they recapitulate the calcifications observed in the cerebral cortex and cerebellum of children with ZIKV-associated encephalopathy; these calcifications are evident in infected mice following Von Kossa staining, as we have previously reported [27]. However, the calcifications do not appear when mice are inoculated during the prenatal phase and evaluated before birth [107,108,109]. The presence of these calcifications could be mediated by the specific expression of GABA-A and NMDA receptor subunits, as their expression and function vary across rodents and humans with age, with equivalences between P10 in rodents and the end of the human prenatal period [74].

The likely mechanism by which GABA blocks cell proliferation could be attributed to the influx of Ca^2+^, as GABA exerts a depolarizing effect. Variations in Ca^2+^ levels affect the phases of the cell cycle, as these phases are highly sensitive to changes in Ca^2+^ concentration [110]. Furthermore, in some studies of neurons subjected to damage, it has been found that the addition of GABA or its agonist muscimol increases calcium flux following glutamate receptor blockade compared to the control group [111].

We observed severe damage to the cerebellar layers and folia in infected mice, with massive loss of Purkinje neurons and, simultaneously, increased GABA immunoreactivity in the remaining neurons (Figure 4k,l). This dysfunction or massive loss of Purkinje neurons was accompanied by loss of the outer and inner granular layers, two layers of glutamate-positive cells that have also been shown to exhibit alterations in previous studies by our research group and other ZIKV infection models [27,112].

In addition to mediating the migration of cells from the external granular layer to the internal granular layer, Purkinje cells make contact with the parallel fibers of cerebellar granule cells to regulate the balance between excitation and inhibition; thus, an imbalance between GABA and glutamate would affect the transmission of information to the deep cerebellar nuclei [113,114]. The loss of this connection to the deep nuclei inhibits the cerebellum’s ability to respond to the information it receives via mossy fibers and climbing fibers, and all of this contributes to the cerebellar neurodegeneration commonly reported in models of ataxia and ZIKV infection [114,115].

Morphological and biochemical changes in the cerebellum may have played a significant role in some of the signs observed in mice infected with the ZIKV, as weight loss is closely associated with dysphagia, which has been reported in up to 70% of cases of ZIKV-associated cerebellar ataxia (CZS) [106]. This condition, along with tremor and uncoordinated gait, is part of the set of signs associated with cerebellar ataxia, which has also been reported during ZIKV infection [114,116].

Strong evidence links cerebellar damage to motor impairments. However, it does not rule out the involvement of the cerebral cortex. GABA and glutamate also showed alterations in the motor cortex, Figure 4a–d and Figure 5a–d. This area shows no changes in the distribution of cortical layers but shows abundant extravasations in the groups of infected mice, Figure 4c,d and Figure 5c,d.

### 3.6. Mechanism of ZIKV Neuropathogenesis Associated with Modifications in the GABA and Glutamate Systems

The results obtained in this manuscript have increased our interest in the role of GABA-A and NMDA receptors as potential viral binding targets. Currently, our research group is conducting molecular docking and molecular dynamics studies between these receptors and one of the ZIKV viral proteins. To date, we have demonstrated that these receptors bind to viral proteins at synaptic contact points or neurotransmitter binding sites (unpublished results).

The various results and analyses obtained from this study indicate that the GABA–glutamate systems modify the synthesis of their neurotransmitters, the expression of the enzymes involved in this process, and their transporters to promote an immunosuppressive environment that could lead to premature differentiation and facilitate viral replication, thanks to increased GABA and the loss of glutamate, the latter being a neurotransmitter associated with the initiation of inflammatory cascades in the presence of a pathogen.

The associations identified from our results allow us to propose the GABAergic system as responsible for the possible excitotoxic effects associated with ZIKV pathogenesis and, therefore, suggest the need to investigate the use of agonists and antagonists of this system on viral load and the improvement of morphological changes in ZIKV infection models through computational approaches and in vitro and in vivo studies.

**Figure 6:**
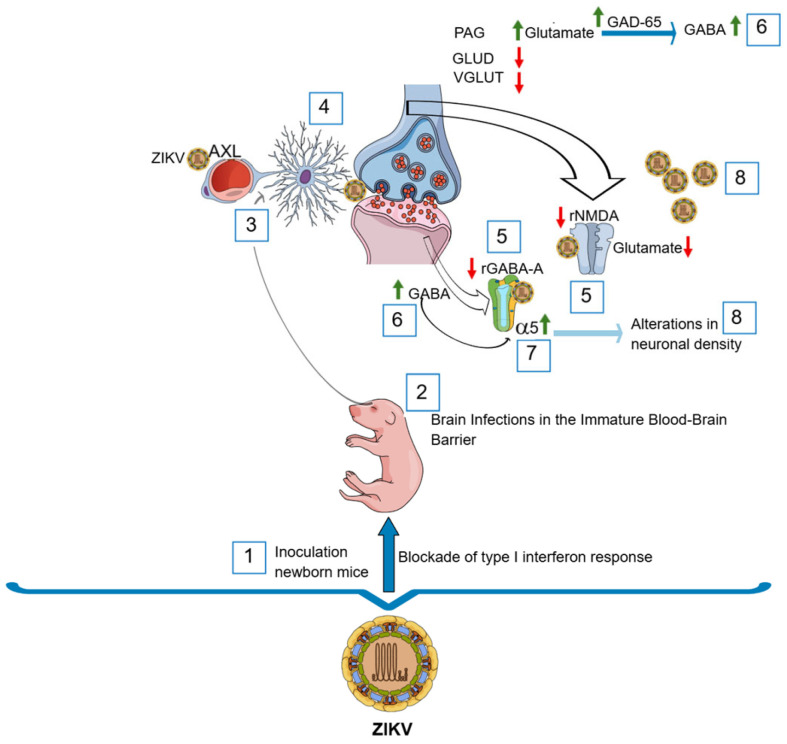
Mechanism of ZIKV neuropathogenesis associated with alterations in GABA and glutamate. This figure summarizes findings on ZIKV-induced changes in components of the GABAergic and glutamatergic systems in the CNS. The effect on the neurotransmitter synthesis pathway is shown in the graphical abstract. Diagram created in Mind the Graph.

Figure 6 summarizes the possible mechanisms of Zika virus (ZIKV) neuropathogenesis associated with changes in the GABA and glutamate systems. This mechanism can be summarized as follows: (1) Viral inoculation in neonatal mice at 0–1 dpi and evasion of the antiviral response due to inefficient blocking of interferon I [96,97]. (2) Neuroinvasion through the immature blood–brain barrier [101,109]. (3) Arrival at astrocyte feet via AXL receptors [11,24]. (4) Viral spread to other glial cells and neurons at the tripartite synapse via (5). Loss of expression of GABA-A and NMDA receptors, associated with binding to viral proteins. (6) Increase in GABA synthesis and decrease in the availability of glutamate as a neurotransmitter [75]. (7) Increase expression of alpha 5 due to extrasynaptic sequestration. (8) Alterations in neuronal proliferation or differentiation and increased viral load as a result of an environment that enhances viral fitness [60,61,68] The increase in PAG, accompanied by the loss of GLUD and the glutamate transporter VGLUT, suggests that during ZIKV infection, glutamate synthesis may temporarily increase its metabolic role as a GABA intermediate but not in its neurotransmitter role [55].

## 4. Materials and Methods

### 4.1. Ethical and Biosafety Considerations

The experimental procedures involving the use of animals were approved by the Institutional Committee for the Care and Use of Laboratory Animals of the National Institute of Health, Bogotá, Colombia (Minute No. 11, dated November 2022; protocol code R-009-2022).

The technical standards were followed as per INS Colombia for laboratory animals that enter the country [117,118] and international guidelines on the care and use of laboratory animals [119,120]. The biosafety and pathogen management standards of the INS and the Biosafety in Microbiological and Biomedical Laboratories (BMBL) of the Center for Disease Control-National Institutes of Health (CDC-NIH) for the management of ZIKV were also followed [120]. All procedures were performed with the support and accompaniment of the veterinarians of the vivarium of the INS.

### 4.2. Viral Stock Generation

VERO E6 cells (ATCC CCL-81, Manassas, VA, USA) were cultured in minimal essential medium (Gibco, Grand Island, NY, USA) supplemented with 1% HEPES (Bleiswijk, The Netherlands), sodium bicarbonate and 2% fetal bovine serum (Gibco, Grand Island, NY, USA). Next, these cells were infected with the ZIKV strain (Zika_virus_459148_Meta_Colombia_2016; GenBank: MH544701.2), which was isolated from a pregnant patient diagnosed with ZIKV during the Colombian epidemic [121]. Five days post-infection, the supernatants were collected, centrifuged at 300× *g* for 5 min and filtered through a 0.2 μm membrane to remove cell debris. Afterward, the supernatants were cryopreserved at −80 °C until further use. Plaque assays were performed with seven serial dilutions (10-fold) to determine viral titers, expressed as plaque-forming units (PFU).

### 4.3. Animals and Viral Inoculation

BALB/c mice of both sexes were kept in the Vivarium of the National Institute of Health, Bogota, Colombia, which has colonies of this strain from the Charles River Laboratory (Charles River Laboratories, Wilmington, MA, USA). The mice were kept in cages with a floor surface of 516 cm^2^ and an interior height of 20 cm (Zyfone^TM^, Lab Products Inc., Seaford, DE, USA) under controlled temperature conditions (23 °C +/− 1 °C) and humidity (55% +/− 10%), and they had access to food and water ad libitum.

From these mice, four litters of neonates between 0 and 1 postnatal day were used, and each litter contained between 5 and 7 mice on average. Two of the litters were inoculated intraperitoneally with 40 microliters of 7.17 × 10^5^ plaque-forming units (PFU) of ZIKV MH544701.2, according to a ZIKV infection model previously obtained by our group [27,121,122,123]. The other two litters were used as control or mock groups and were inoculated with the same volume but with a supernatant solution of uninfected VERO cells.

From the inoculated litters, one group of 10 mice (5 infected and 5 mock) was used for differential expression assays by q-RT-PCR of GABA-A and NMDA receptors, and for enzymes involved in GABA synthesis and transport, for Western blotting assays (first group). The other two litters (5 infected and 5 mock) were used for immunohistochemical assays of the levels of GABA and glutamate neurotransmitters in the cortex and cerebellum of mice infected with ZIKV, which were distributed in the same way as those in the control group and the infected group (second group). Control and infected animals were kept in separate cages until the end of the experiment.

Clinical signs were monitored daily, and on day 10 post-inoculation (dpi), the mice in the first group were subjected to euthanasia with *CO*_2_ to minimize suffering. The brains were removed, the hemispheres were divided, and the cerebral cortex and the cerebellum were separated and stored separately. One of the hemispheres was stored in RNALater^®^ (Sigma Aldrich, Merck, Darmstadt, Germany) for one week, after which the reagent was removed and stored at −80 °C. The second hemisphere was stored fresh at the same temperature for future Western blot assays.

**Figure 7:**
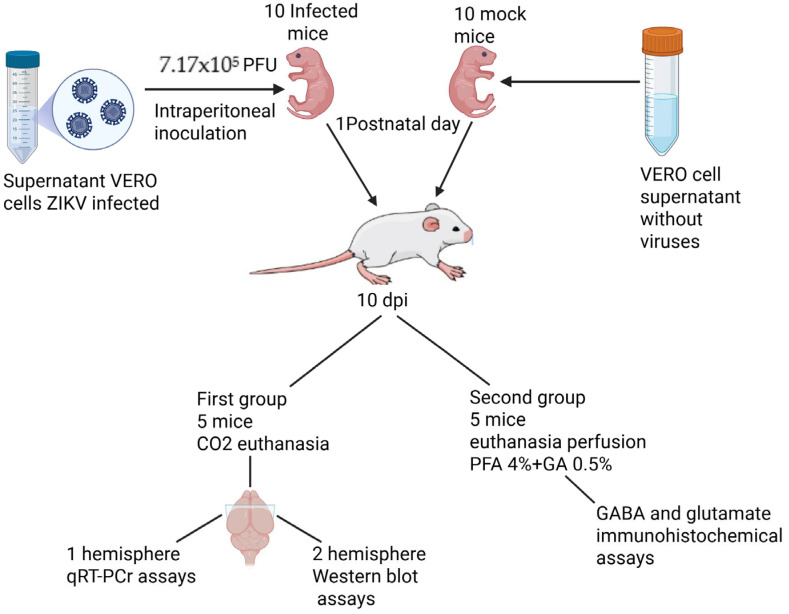
Scheme of the infection groups used and their respective mocks. The animals were inoculated on day 1P and evaluated at 10 dpi. The litters were distributed into two groups of 5 infected animals with the same number of mock mice. Graphic created in BioRender and Mind the Graph.

The mice in the second group used for immunohistochemical assays of neurotransmitters were also subjected to euthanasia on day 10 dpi; however, these mice were anesthetized with a volume of 10–20 microliters of 30% chloral hydrate intraperitoneally for sedation. Next, the mice were perfused intracardially with a mixture of 4% paraformaldehyde-0.5% glutaraldehyde (PFA4%;–GA0.5%). The brains were extracted and stored in the same solution for six hours and then in 4% PFA until further use. Figure 7 shows a diagram illustrating the distribution of the groups.

To ensure animal welfare and optimize the use of experimental subjects, the mice evaluated in this study belong to the same experimental cohort previously described by our group [24]. Since these were suckling pups, they remained with their mothers in separate cages—for control and infected groups—and the mothers were provided with food and water ad libitum. Daily weight measurements were taken for both control and infected mice, and clinical signs of disease associated with the infection were monitored, as established and reported in our previous characterization of this cohort [24].

### 4.4. Detection of ZIKV

Total RNA was extracted from the cortex and cerebellum using the miRNeasy^®^ Mini kit (Qiagen, Hilden, Germany) following the manufacturer’s instructions. The presence–absence tests were performed following the methodology previously standardized by our group [124]. A one-step RT—PCR kit SuperScript^®^ III (Invitrogen™ Life Technologies, Carlsbad, CA, USA) and the following reaction conditions were used: 0.42 mM of each forward and reverse primer and 0.3 mM probe for ZIKV FAM-BHQ-1 with 1.5 μL of RNA for a total volume of 10 μL for each reaction. All samples were evaluated in duplicate in an Applied Biosystems 7500 Fast Dx Real-time PCR thermal cycler with the following reaction conditions: reverse transcription at 50 °C for 15 min, enzyme activation at 95 °C for 2 min, and 45 cycles of 95 °C for 15 s each and 55 °C for 45 s for hybridization and extension. In these assays, the number of viral copies in the cortex and cerebellum was determined using a viral RNA construct generated by our group [124]. For these assays, the Ct values obtained by viral detection PCR were interpolated using the standard curve of the RNA construct of known concentration. The final value is expressed as the number of viral copies/nanogram of total RNA [27].

### 4.5. Differential Expression Assays for GABA-A and Glutamate Receptor Subunits

For the mRNA differential expression assays, RNA integrity measurements were performed by measuring the RIN in an Agilent TapeStation 4200 reference 5301 (Stevens Creek Blvd., Santa Clara, CA, USA). The samples with the best RINs were used (Figures in Supplement S7), and qRT–PCR differential expression assays were performed. The mRNA levels of the subunits of the GABA-A and NMDA receptors present in the cortex and cerebellum of the mouse that were expressed until the second postnatal week were evaluated according to the experimental window of the infection model. The subunits were determined according to Allen’s atlas (http://developingmouse.brain-map.org, accessed 15 November 2021.), Table 1 and Table 2 and those reported in the literature [125,126]. The primers were designed with Primer Select software (Lasergene version 7.2.1) or as reported in the literature, and those that passed the thermal stability tests were chosen according to the Beacon designer software and OligoAnalyzer (https://www.idtdna.com/pages/tools/oligoanalyzer, accessed 7 December 2022) (Table 3 and Table 4).

Prior to the differential expression assays, standard curves were constructed to determine the dynamic ranges of expression and the efficiency of the reaction. The amount of nanograms of RNA to be used for each gene to be evaluated was determined using the software Applied Biosystems 7500 v2.0.6. The dynamic range of optimal concentrations was determined in the groups of genes evaluated for use with the Luna Universal qPCR Master Mix system kit (New England Biolab, Ipswich, MI, USA), which uses Sybr Green as the only detection probe. In all the assays, GAPDH was used as a reference gene, with four biological replicates (four control and infected animals) and three technical replicates (replicates).

The data were analyzed using the Livak method [53] and the efficiency per run was calculated using the software LinReg PCR (2014.x) [127]. To estimate the relative gene expression, the software ExpressionSuite v 1.1 and the Gene Expression Ct’s Difference (GED) were used [128].

**Table 3:** Primer sequences for differential expression assays of GABA-A receptors.

Gene Symbol	Primer Sequence (5′–3′)		MT (°C)	Amplicon Size (bp)	GenBank Accession no.	Author
Gabra1	S:	AAAAGCGTGGTTCCAGAAAA	**	84	**	Mendu et al. [129]
	A:	GCTGGTTGCTGTAGGAGCAT	**			
Gabra2	S:	TTGGGACGGGAAGAGTGTAG	51.6 *	107	NM_008066.4	Design
	A:	AAAGATTCGGGGCATAGTTG	51 *			
Gabra3	S:	AGTCAGCCCCTCAGGAAAGTAGTG	56.8 *	124	NM_008067.4	Design
	A:	GTAGGCAGCCATGAATCCAAAATA	56.2 *			
Gabra4	S:	AGGTGCCAAAGGAGTCTTCTA	60.2 *	136	NM_010251	Primer bank ID 85861216c2
	A:	TAGCCCATCTTCCGTCTGAGG	62.8 *			
Gabrα5	S:	GCCCTGGAAGCAGCTAAAAT	59 *	227	NM_176942	
	A:	CGGGACATTTTGTCGATCTT	59 *			
Gabra6	S:	CTGAAAGGCAGGCACAAACT	59 *	221	NM_008068	Dvoryanchikov et al. [130]
	A:	TAAGAGCAATGGGGGAGAGA	59 *			
Gabrb1	S:	GGTCACAGTGAAAAACCGAATG	60 *	170	NM_008069	Primer Bank ID 118131022c1
	A:	CGATGTCATCCGTGGTATAGCC	62.3 *			
Gabrb2	S:	GGGTGCCTGACACCTACTTC	61.9 *	86	NM_008070	Primer Bank ID 124301234c3
	A:	GGGATGCAATCGAATCATACGG	61.2 *			
Gabrb3	S:	CACGCTTGACAATCGAGTGG	61.3 *	109	NM_008071	Primer Bank ID 84662777c2
	A:	GCGGATCATGCGGTTTTTCAC	62.7 *			
	A:	ATGAAGTTGAAGGTAGCACTCTG	60 *			
Gabrg2	S:	ACTTCTGGTGACTATGTGGTGAT	**	147	**	Mendu et al. [129]
	A:	GGCAGGAACAGCATCCTTATTG	**			
Gabrg3	S:	ATTACATCCAGATTCCACAAGATG	**	149	**	Mendu et al. [129]
	A:	CACAGGTGTCCTCAAATTCCT	**			
Gabrd	S:	CCAGCATTGACCATATCTCAGAG	60.2 *	190	NM_008072	Primer Bank ID 160707922c2
	A:	TCATGGAACCAGGCAGATTTG	60.3 *			
Gabre	S:	TGGAGGGTTGGACCTCATGTT	62.9 *	110	NM_017369	Primer Bank ID 114145508c1
	A:	GATTCAGGCGGAGTTAGAGGC	62.3 *			
	A:	AGCATGATGTTGTCGGTGGTG	62.9 *			
Gabr3	S:	CAACTCAACAGGAGGGGAAA	**	101	**	Mendu et al. [129]
	A:	TCCACATCAGTCTCGCTGTC	**			

* Annealing temperature, ** Not reported.

**Table 4:** Primer sequences for NMDA receptor differential expression assays.

Gene Symbol	Primer Sequence (5′–3′)		MT (°C)	Amplicon Size (bp)	GenBank Accession no.	Author
GRIN1	S:	TCCCAACGACCACTTCACTC	61.5 *	95	NM_008169	Primer Bank ID 294997255c3
	A:	AGTAGATGGACATTCGGGTAGTC	60.7 *			
GRIN2A	S:	ACGTGACAGAACGCGAACTT	62.3 *	100	NM_008170	Primer Bank ID 41680704c1
	A:	TCAGTGCGGTTCATCAATAACG	60.9 *			
GRIN2B	S:	CAGCAAAGCTCGTTCCCAAAA	61.7 *	163	NM_008171	Primer Bank ID 117168298c1
	A:	GTCAGTCTCGTTCATGGCTAC	60.2 *			
GRIN2C	S:	GCCCTGCTTCTCACTTCACTC	62.7 *	198	NM_010350	Primer Bank ID 602753a1
	A:	GTTGGTATTGTTGACCCCGAT	60 *			
GRIN2D	S:	GAGCCTTCTTGTCATACATCGAG	60.5 *	235	NM_008172	Primer Bank ID 144922605c2
	A:	CCCACCATGAACCAGACGTAG	62.1 *			
GRIN3B	S:	CCAGCGGCAGTAAATTGTGAG	61.6 *	174	NM_130455	Primer Bank ID 53759069c3
	A:	CCTGCGTAAGCTCCATACCTT	61.6 *			

* Annealing temperature.

### 4.6. Evaluation of Effects on GABA—Glutamate Neurotransmitter Systems

The synthesis of the neurotransmitters GABA and glutamate was evaluated in the two groups of mice as described in Section 4.3. In the first group (fresh tissue) and with one of the cerebral hemispheres of the mice, analyses were performed on the expression of molecular components of GABA and Glutamate metabolism and transport by qRT–PCR assays with the same methodology described in Section 4.5 but with the primers described in Table 5. Western blot assays were performed with the other hemisphere according to the methodology described in Section 4.7. In the second group of mice, studies were performed on the immunoreactivity of neurotransmitters (tissues fixed in formalin, according to the methodology described in Section 4.8).

**Table 5:** Primer sequences for differential expression assays of molecular components of GABA and Glutamate metabolism and transport.

Gene	Primer Sequence (5′–3′)		MT (°C)	Amplicon Size	GenBank Accession no.	Author
GAD-65	S:	GTCTCCTGGCTCCGGCTTTTGGTCCTT	68.4 *	117	L16980.1	Design
	A:	CCGATGCCGCCCGTGAACTTTTG	67.6 *			
GAD-67	S:	GGGCTATGTTCCCTTTATGT	60 *	184	AF326547.1	Ma et al. [131]
	A:	CCTTTCTATGCCGCTGAGT	60 *			
VGAT	S:	TGGGAAGCGGGCTGGAACGTGACAAATG	72.5 *	118	AY578289.1	Design
	A:	CCACTGCGGCGAAGATGATGAGGAACAA	69.7 *			
VGLUT	S:	TTGCCGCAGCTGGACCTTCTACCT	64.1 *	102	NM_182993.2	Primer Bank
	A:	GCGCCGACACCAGCCCCACCTT	69.5 *			ID 602753a1
PAG	S:	TTGTCCCCAACGTCATGGGC	57 *	237	NM_001081081.2	Gao et al. [132]
	A:	GGAGGGCAGACACATCTCCA	57 *			
GLUD	S:	GCGGCGCCAGCATCGTAGAGG	65.5 *	146	NM_008133.4	Primer Bank
	A:	CGCCGGATGGGGAAGGAGAGG	65.4 *			ID 53759069c3

* Annealing temperature.

### 4.7. Expression Assays of Molecular Components of GABA and Glutamate Metabolism and Transport

The analysis for these molecules was performed on extracts of proteins from the cortex and cerebellum of mice infected with ZIKV and their respective mock controls. A mixture of 1 mL of N-PER (ref 87792) and 40 mL of protease inhibitor (ref 78842) (Thermo Fisher Scientific, Rockford, IL, USA) was used as the lysis buffer. One milligram of cortex and cerebellum tissue was weighed separately in 1.5 mL Eppendorf tubes. In each tube, 300 μL of the lysis buffer mixture was homogenized using a pellet pestle. Another 200 μL was added for a second homogenization, and the volume was brought up to 1 mL with lysis buffer. The samples were then centrifuged at 10,000× *g* for 10 min at 4 °C. The supernatants were transferred to new tubes, and several of the tubes were aliquoted in volumes of 100 μL and stored at −80 °C until use.

Prior to the Western blot assays, the proteins extracted by the bicinchoninic acid method were quantified [133], using the Perce ^TM^ BCA Protein Assay Kit (ref. 23327). A volume of each sample was taken to ensure a concentration of 10 mg per sample according to the standardizations previously performed. Afterward, the proteins were denatured using a 2X Novex Tris-Glycine SDS kit using the reactive sample buffer as loading buffer (ref. LC2676) and 10X Nupage Sample reducing agent as the reducing agent (ref NP009) (Thermo Fisher Scientific Rockford, IL, USA). Each reagent was maintained at 1x proportions per reaction mixture. The denaturation conditions are described in Table 6.

After denaturation, the samples were subjected to SDS–PAGE electrophoresis, a tris glycine system with 4% gels for the stacking gene and a 10% gel for the resolving gel from the reagent 29:1 acrylamide-bisacrylamide (ref HC2040; Invitrogen; Thermo Fisher Scientific, Rockford, IL, USA). The electrophoresis was run for 50 min at 50 V (stacking gel) and 140 min at 100 V (resolving gel) at room temperature. Protein transfer was performed with PVDF membranes (Ref. 88518; Thermo Fisher Scientific, Rockford, IL, USA) in a humid chamber at 100 V for 1 h and 30 min or overnight at 30 V for 16 h. Following the transfer, the No-StainTM total protein detection kit was used as a loading control. The protein labeling reagent was from Thermo Fisher Scientific (Rockford, IL, USA; ref. A44449), and this labeling was visualized using a ChemiDoc ^TM^ MP Imaging System through the channels enabled for the fluorophores Alexa 488 or Alexa 546.

After total protein was developed, the membranes were blocked by incubation with 5% skim milk for one hour and incubated with primary antibody overnight at the concentrations described in Table 6. The membranes were subsequently washed 5 times for 5 min in Tris saline-Tween buffer (1× TBST) at pH 7.5 and incubated for two hours in secondaries coupled to horseradish peroxidase (HRP)-conjugated anti-mouse (7076 S; Cell Signaling; Danvers, MA, USA) and anti-rabbit (7074 S; Cell Signaling) according to the species of the primary. Finally, the membranes were visualized by chemiluminescence using a SuperSignal ^TM^ kit. West Pico PLUS Chemiluminescent Substrate (Thermo Fisher Scientific, Rockford, IL, USA; ref. 34578) was used, and the total protein was photographed. The images obtained were analyzed using *Image Lab* 6.1 software, and the data were normalized to the measurements of total protein loaded following the recommendations of Taylor et al. [54].

### 4.8. Immunohistochemical Assays for ZIKV, GABA and Glutamate

The second group of mice described in Section 4.3 was anesthetized at 10 dpi by intraperitoneal injection of 0.02 mL of 30% chloral hydrate. When anesthesia was achieved, intracardiac perfusion was performed initially with phosphate-buffered saline solution (PBS) at pH 7.3 for 5 min and then with a solution composed of two fixatives, paraformaldehyde (PFA) at 4% and glutaraldehyde (GA) at 0.5% according to previously standardized tests [32]. Once the perfusion was completed, each brain was extracted, avoiding damage to the brain tissue. Next, the whole brains were submerged in a fixing solution of the same composition as that used in the perfusion and remained in the solution for 6 to 24 h at 4 °C. Afterward, the brains were transferred to a 4% PFA solution at 4 °C until the sections were prepared for immunohistochemical analysis.

In the fixed brains, the cerebral cortex was separated from the cerebellum, and 30-micron sections were made on a vibratome (Vibratome^®^) (Leica, VT1000 S, Wetzlar, Germany) in the sagittal plane for the cerebellum and in the coronal plane for the cerebral cortex. The sections were processed in suspension in glass dishes (Mini-Petri) with a volume of 1.25 mL in all immunodetection steps, and the entire process was performed at room temperature under constant stirring. A wash was performed in phosphate-buffered saline (PBS) at pH 7.3 after each of the reactions. The sections were incubated for half an hour under agitation in 0.5% borohydride Merck Millipore 16940-66-2 (San Jose, CA, USA) for the blocking of aldehydes, after which they were incubated in 3% hydrogen peroxide for the inactivation of endogenous peroxidase and in blocking solution containing normal serum, bovine serum albumin (BSA) and Triton, prior to incubation with primary polyclonal rabbit antibodies (Antiglutamate Sigma G6642 and anti-GABA Sigma A2052) overnight at 1:2000 dilutions (St. Louis, MO, USA). Three washes were performed with BS 1X for 5 min. The sections were incubated with an anti-rabbit secondary antibody (Sigma-Aldrich B8895). Washes were performed again with PBS, after which the sections were developed with developer or DAB-Ni (Vector Laboratories, Inc., 6737 Mowry Ave Newark, CA, USA), subjected to new washes with 1X PBS and mounted with Entellan (Sigma Aldrich, Merck, Darmstadt, Germany; ref 107961). In all the brains, the presence of viral antigen was verified with an anti-ZIKV antibody donated by the CDC, according to the methodology previously reported by our group [27].

### 4.9. Histological and Digital Analysis of the Images

For the quantitative analysis of the immunohistochemical assays of GABA and glutamate, neuron counts were performed in the cortex samples of control and infected animals using a Zeiss Netzmiier mesh micrometer of 1 mm^2^ or a digital grid of the Micrometrics program according to previous studies [27,33]. The counts were performed by exploring specific fields with the objective of 10x and since the size of the cortices varied between the motor and the somatosensory performed area approximations using the counting grid. To facilitate counting, Layers V and VI of the motor cortex were divided from (a) to (d) and those from the somatosensory cortex from (a) to (c).

The analyses of the cerebellum focused on the folia of the cerebellar cortex; the images were digitized with the aid of an Axiophot Zeiss microscope and an Evolution VF- camera and Qcapture Pro 6.0 software. Densitometric analyses (light transmittance measurements) of three cerebellar folia were performed in one section per sample with the help of the ImageJ 1.54g program (National Institutes of Health, Bethesda, MD, USA). Glutamate measurements were performed in the granular layer, and measurements for GABA were carried out in the granular layer and Purkinje cells.

### 4.10. Statistical Analysis and Illustrations

For statistical analyses, hypothesis testing was performed using the parametric Student’s *t*-test for data with a normal distribution based on bias and kurtosis, normality tests, goodness-of-fit tests, and homoscedasticity tests. Data that did not meet these criteria were analyzed using the nonparametric Mann–Whitney *U* test in Statgraphics Centurion XVI version 16.1.03 (Statgraphics Technologies, Inc., The Plains, VA, USA). The graphs and illustrations within the figures were created using GraphPad Prism version 10.6.1 (GraphPad Software, Boston, MA, USA), Adobe Illustrator 1.0 (Adobe Inc., San Jose, CA, USA), and BioRender.com (Toronto, ON, Canada).

For histological analysis, including cell counts (discrete data) and optical density measurements (continuous data), the non-parametric Wilcoxon–Mann–Whitney U test was used. This approach was selected to provide a robust and conservative comparison without assuming normality, given that both continuous and discrete data were being compared. Statistical analyses of these parameters were performed using InfoStat software (version 2020; National University of Córdoba, Argentina). This methodology is consistent with our established laboratory protocols and previously published studies [32,134,135], following standard biostatistical recommendations [136]. Data are presented as mean ± standard deviation (SD) for the groups.

## 5. Conclusions

Our results indicate that, in the final phase of ZIKV infection, there may be a loss of glutamate and an increase in GABA synthesis, thereby generating neurotransmitter dysfunction and a metabolic environment that favors viral replication, considering that the increase in GABA and the loss of glutamate have been associated with immunosuppressive effects for flaviviruses such as JEV.

The increase in GABA immunoreactivity in the cerebellum could be associated with the observed increase in α5 subunit expression following sequestration of GABA-A receptor proteins in the extrasynaptic space. This increase could contribute to defects in neuronal migration and synapses in tissues infected with ZIKV. Our results highlight the need to evaluate glutamatergic and GABAergic agonists as key candidates for potential antiviral agents or neuroprotective targets in models of ZIKV infection.

## Figures and Tables

**Table 1:** Subunits of GABA-A receptors present in the second postnatal week of the mouse.

Name of the Gene	Name of the Subunit	Area of Expression
Gabra1	α1	cortex and cerebellum
Gabra2	α2	cortex and cerebellum
Gabra3	α3	cortex and cerebellum
Gabra4	α4	cortex
Gabrα5	α5	cortex and cerebellum
Gabra6	α6	cerebellum
Gabrb1	β1	cortex and cerebellum
Gabrb2	β2	cortex and cerebellum
Gabrb3	β3	cortex and cerebellum
Gabrg2	γ2	cortex and cerebellum
Gabrg3	γ3	cortex
Gabrd	δ	cerebellum
Gabre	ε	cerebellum
Gabr3	ρ3	cerebellum

**Table 2:** NMDAR subunits present in the second postnatal week of the mouse.

Name of the Gene	Name of the Subunit	Area of Expression
GRIN1	GluN1	cortex and cerebellum
GRIN2A	GluN2A	cortex and cerebellum
GRIN2B	GluN2B	cortex and cerebellum
GRIN2C	GluN2C	cortex and cerebellum
GRIN3B	GluN3B	cortex and cerebellum

**Table 6:** Antibodies used for Western blot assays and working conditions.

Antibody	Brand	Reference	Observed Molecular Weight (kDa)	Type of Antibody	Blocking and Denaturation Conditions	Primary Incubation	Secondary Incubation
GLUD1/2 (C-10)	SANTA CRUZ BIOTECHNOLOGY, INC.	sc-515542	50–55	Mouse monoclonal IgG1 κ	* Milk 5%	[1:2000] milk 5% 4 °C/Overnight	Anti-mouse (HRP) [1:5000] milk 5% 4 °C/Overnight
GAD65 antibody [GAD-6]	ABclonal	a22728	65	Mouse	* Milk 5%	[1:10,000] milk 5% 4 °C/Overnight	Anti-mouse (HRP) [1:5000] milk 5% 4 °C/Overnight
VGluT1 antibody [EPR22269]	ABCAM	ab227805	62	Rabbit monoclonal [EPR22269] to VGluT1	* Milk 5%	[1:4000]	Anti-rabbit (HRP) [1:5000]
VGAT (F-2)	ABclonal.	Slc32A1	57	Monoclonal mouse	* Milk 5%	[1:500]	Anti-mouse (HRP) [1:5000] milk 5% 4 °C/Overnight
Anti-GAD67 antibody [K-87]	ABCAM	ab26116	67	Monoclonal mouse	* Milk 5%	[1:2000] milk 5% 4 °C Overnight	Anti-mouse (HRP) [1:5000] milk 5% 4 °C/Overnight
Recombinant Anti-Glutaminase C antibody (PAG)	ABCAM	ab202027	65	Monoclonal rabbit	* Milk 5%	[1:15,000] cortex; [1:5000] cerebellum	Anti-rabbit (HRP) [1:5000] milk 5% 4 °C/Overnight

* Non-fat dry milk.

## Data Availability

https://data.mendeley.com/datasets/2wr5ghgj5c/1 (accessed on 7 January 2026).

## Supplementary Materials

The following supporting information can be downloaded at https://www.mdpi.com/article/10.3390/ijms27114833/s1.
