# Supplementary material for: Alterations in the Components of the GABA–Glutamate System During ZIKV Infection: A Neuroscience Approach
Source: Int J Mol Sci. 2026 May 27;27(11):4833. doi: 10.3390/ijms27114833 (PMC13256588; doi:10.3390/ijms27114833)
Supplement: Supplementary file 1 [file ijms-27-04833-s001.zip › Supplement 1. Efficiencies and fold of change GABA-A and NMDA.pdf]

Supplement 1. Efficiencies and fold changes GABA-A and NMDA receptors

Table S1.1 GABA-A cerebral cortex

Brain area	Gen name	Fold change ZIKV	SD Mock-infected		Reaction efficiency	P value
			Mock	Infected		
Cerebral cortex	Gabra 1	0,421	0,293	0,391	93,8	0,000082*
	Gabra 2	0,315	0,168	0,808	100,8	2,15113E-10**
	Gabra 3	0,57	0,249	0,036	92,5	0,00033*
	Beta 1	0,78	0,082	0,088	84,5	5,44069E-9**
	Beta 2	0,83	0,066	0,126	83,6	0,000078*
	Beta 3	0,78	0,062	0,144	83,6	4,52423E-9**
	Gabrg2	0,928	0,092	0,08	87,7	0,0041*
	Gabrg3	0,73	0,088	0,146	88,7	8,14111E-10**
	Gabra4	0,747	0,028	0,186	84,6	0,0111092*
	Gabra5	0,804	0,085	0,133	75,5	6,12636E-7**

Note: The data obtained for mock and ZIKV groups for each marker were compared using the Wilcoxon-Mann-Whitney U test (*) and the Student's t-test (**) from the results obtained to determine the normality criteria. The data correspond to the analysis of four biological samples and three technical replicates.

Table S1.2 GABA-A cerebellum

Brain area	Gen name	Fold change ZIKV	SD Mock-infected		Reaction efficiency	P value
			Mock	Infected		
**Cerebellum**	Gabra1	0,913	0,09	0,156	85,1	0,056**
	Gabra3	1,027	0,398	0,264	98,9	0,946**
	Grabra5	1,309	0,45	0,135	91,7	0,035**
	Gabra6	0,74	0,239	0,555	87	0,008**
	Gabrb1	0,639	0,9	0,368	90,2	0,032**
	Gabrb2	0,951	0,131	0,099	83,1	0,6318**
	Gabrb3	0,821	0,214	0,202	90,2	0,0014**
	Grabg2	0,83	0,213	0,246	89,1	0,013**
	Gabrd	0,916	0,305	0,421	90,1	0,688**
	Gabre	1,786	0,842	0,439	86	0,022**
	Gabrr3	0,662	1,486	0,758	97,1	0,8066**

Table S1.3 NMDA cerebral cortex

Brain area	Gen name	Fold change ZIKV	SD Mock-infected		Reaction efficiency	P value
			Mock	Infected		
Cerebral cortex	GRIN1	0,815	0,086	0,756	86,8	0,00128468**
	GRIN2A	0,625	0,127	0,64	91,7	0,007*
	GRIN2B	0,634	0,14	0,507	84,3	0,0005**
	GRIN3B	0,79	1,185	2,384	85,7	0,320912**

Table S1.4 NMDA cerebellum

Brain area	Gen name	Fold change	SD Mock-infected		Reaction efficiency	P value
		ZIKV	Mock	Infected		
Cerebellum	GRIN1	0,751	0,134	0,271	86,1	0,002*
	GRIN2A	0,651	0,094	0,347	88,9	0,002*
	GRIN2B	0,427	0,108	0,448	67	0,00006*
	GRIN2C	0,97	6,285	0,504	62,1	0,961003**
	GRIN3B	0,429	0,429	0,536	82,7	0,004049
