# Supplementary material for: Alterations in the Components of the GABA–Glutamate System During ZIKV Infection: A Neuroscience Approach
Source: Int J Mol Sci. 2026 May 27;27(11):4833. doi: 10.3390/ijms27114833 (PMC13256588; doi:10.3390/ijms27114833)
Supplement: Supplementary file 1 [file ijms-27-04833-s001.zip › Supplement 2. qRT-PCR Efficiencies and Fold Changes for Molecular Components of GABA and Glutamate Metabolism and Transport.pdf]

Supplement 2. qRT-PCR Efficiencies and Fold Changes for Molecular Components of GABA and Glutamate Metabolism and Transport.

Table S2.1. Cerebral cortex

**Brain area**	**Gen name**	**Fold change infected**	**SD**		**Reaction efficiency**	P value
			**Mock**	**Infected**		
Cerebral cortex	GAD-65	0,628	0,241	0,607	92,8	0,0024535**
	GAD-67	1,659	0,55	0,307	94,7	0,00102213**
	PAG	0,391	0,073	0,123	89,9	<<0**
	GLUD	0,556	0,08	0,271	96	0,000003*
	VGAT	0,69	0,227	0,584	85,1	0,037**
	Vglut	0,797	0,234	0,097	95,6	0,0012*

Table S2.2 Cerebellum

Brain area	Gen name	Fold change infected	SD		Reaction efficiency	P value
			Mock	Infectados		
Cerebelo	GAD-65	1,19	0,151	0,337	92,9	0,652039**
	GAD-67	1,097	0,2688	0,403	91,6	0,22373*
	PAG	0,314	0,121	0,314	89,2	<< 0**
	GLUD	0,722	0,123	0,234	96,3	0,0000016**
	VGAT	0,993	0,128	0,478	86,8	0,498736*
	Vglut	1,157	0,205	0,218	92,5	0,0097**

Note: The data obtained for mock and ZIKV groups for each marker were compared using the Wilcoxon-Mann-Whitney U test (*) and the Student's t-test (**) from the results obtained to determine the normality criteria. The data correspond to the analysis of four biological samples and three technical replicates.
