# Supplementary material for: Alterations in the Components of the GABA–Glutamate System During ZIKV Infection: A Neuroscience Approach
Source: Int J Mol Sci. 2026 May 27;27(11):4833. doi: 10.3390/ijms27114833 (PMC13256588; doi:10.3390/ijms27114833)
Supplement: Supplementary file 1 [file ijms-27-04833-s001.zip › Supplement 3. Total Protein Loading Control Images.pdf]

Supplement 3. Total Protein Loading Control Images (Cerebral Cortex and Cerebellum)

Cerebral cortex

Membrane 1 total protein	Membrane 2 total protein
GAD-65	GAD-65
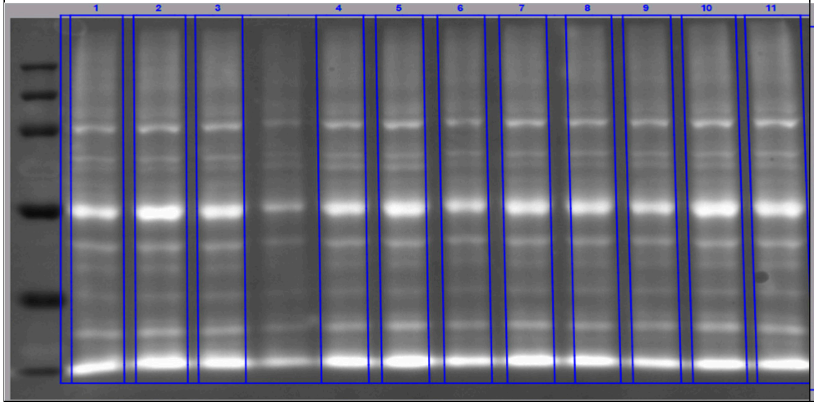	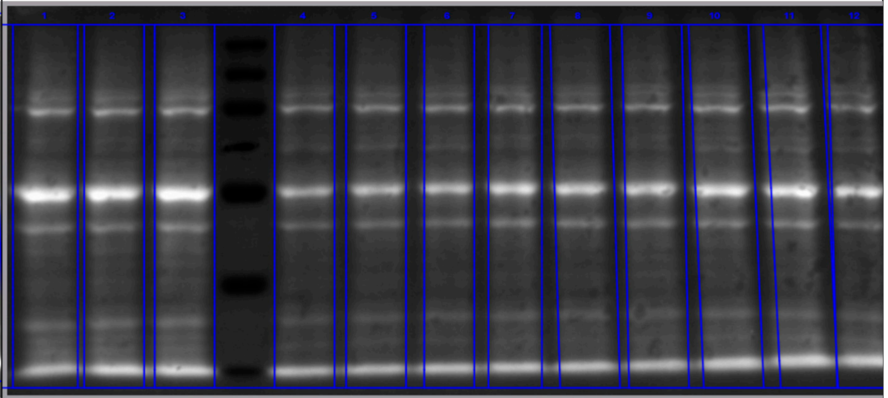
GAD-67	GAD-67
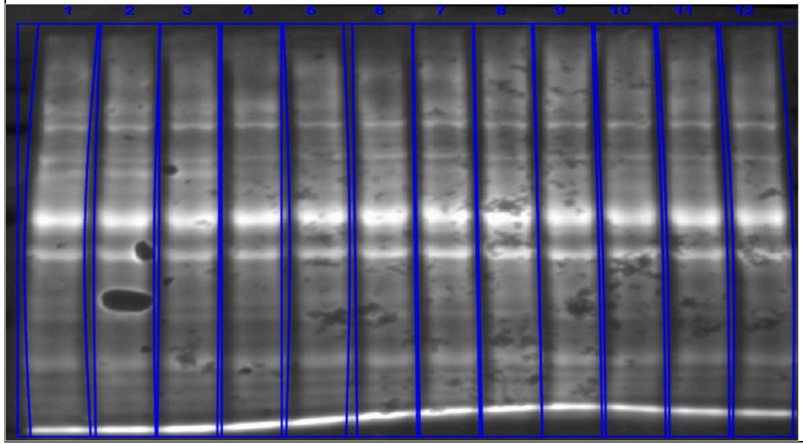	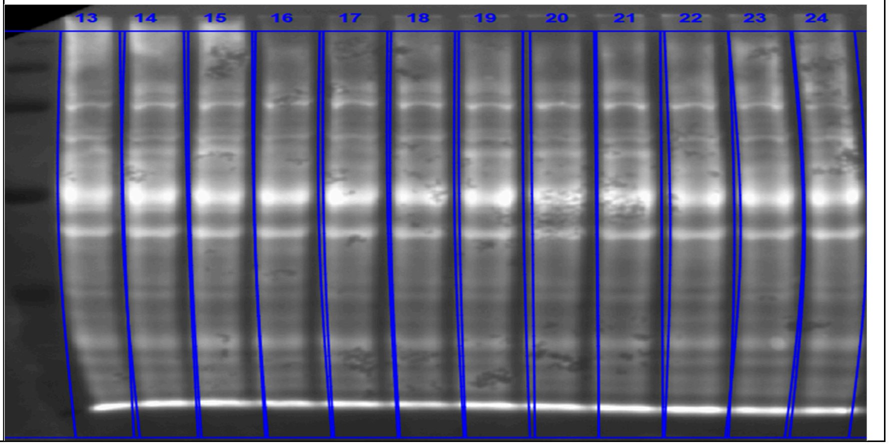

Membrane 1 total protein	Membrane 2 total protein
PAG 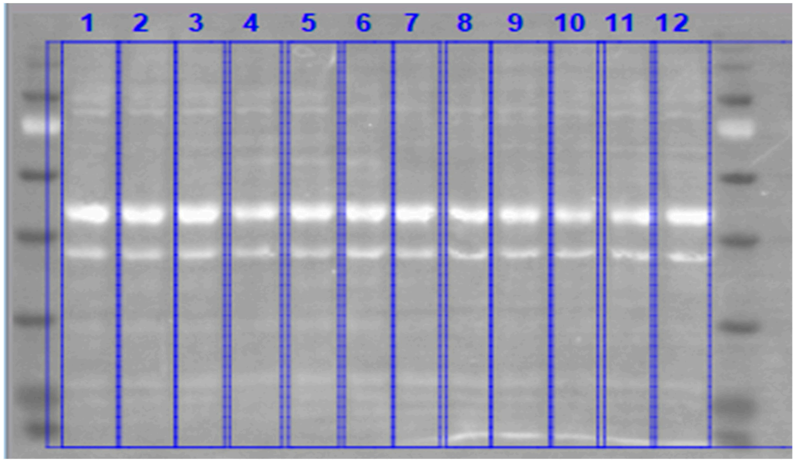	PAG 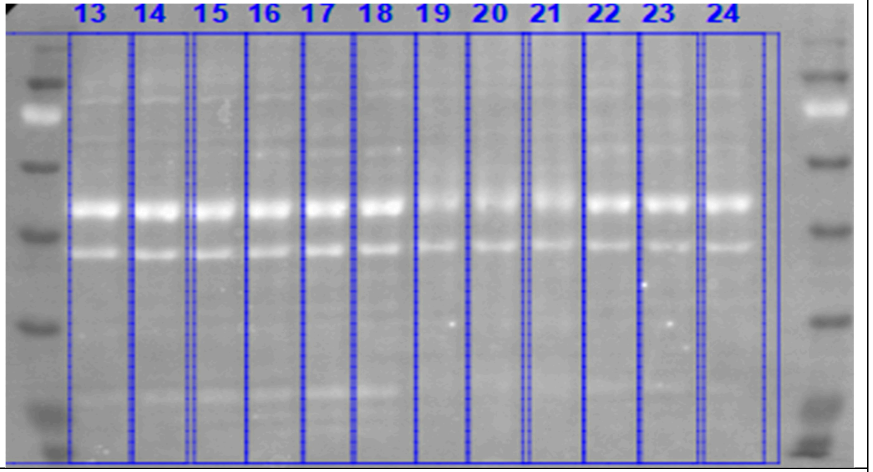
GLUD 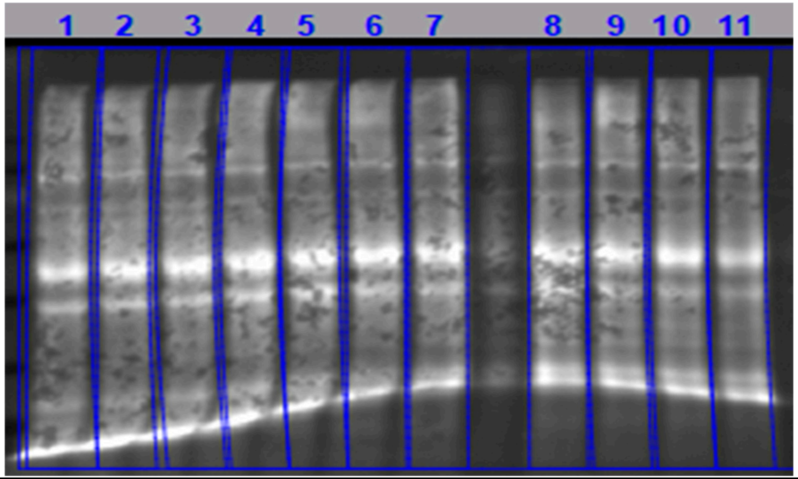	GLUD 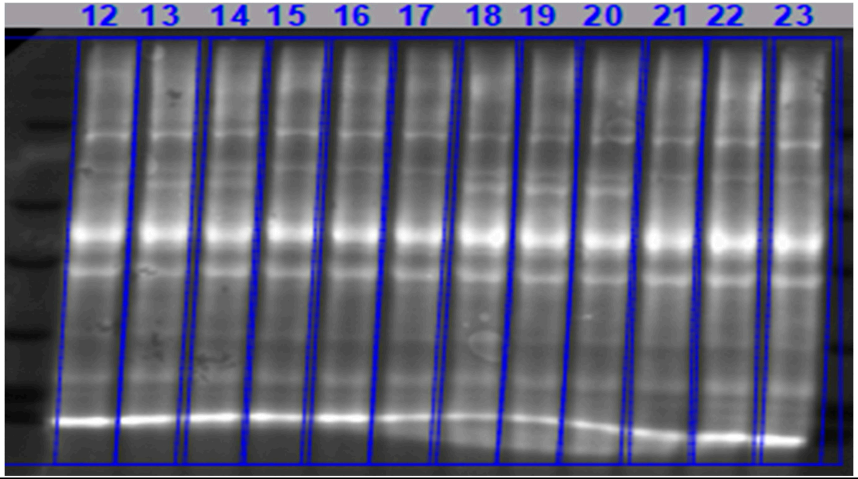

Membrane 1 total protein	Membrane 1 total protein
VGAT<br>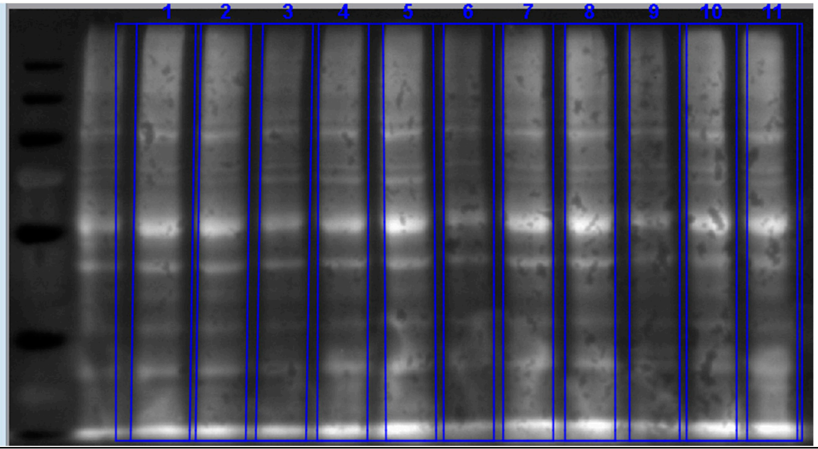	VGAT<br>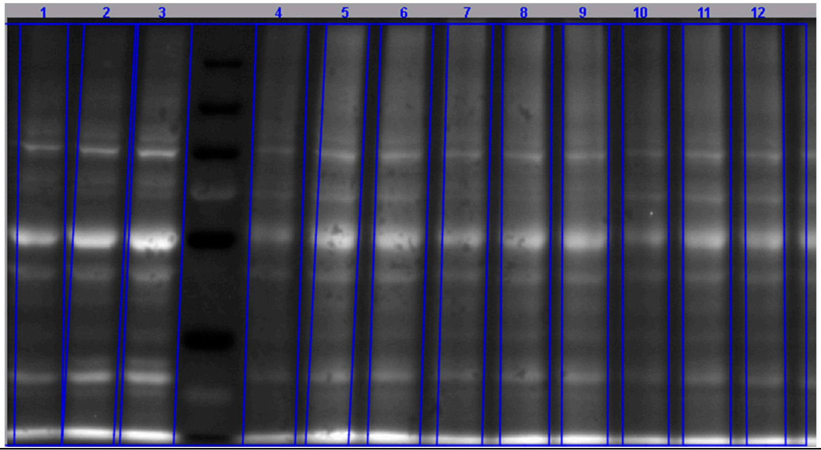
VGLUT<br>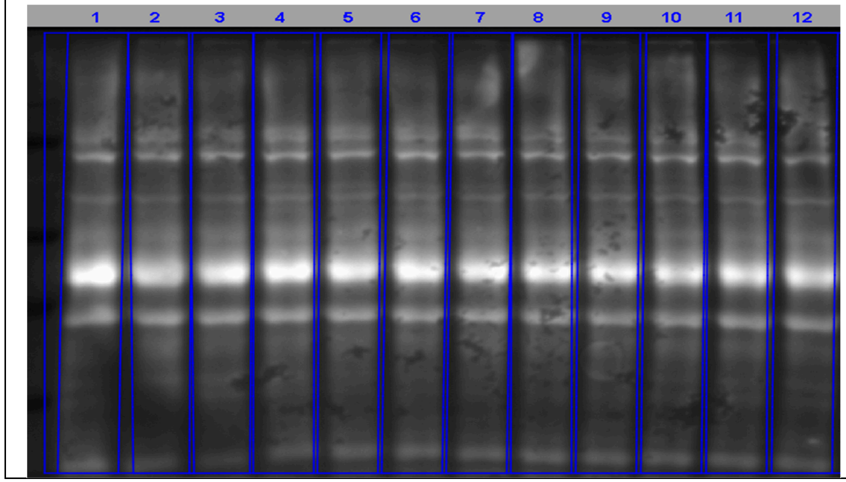	VGLUT<br>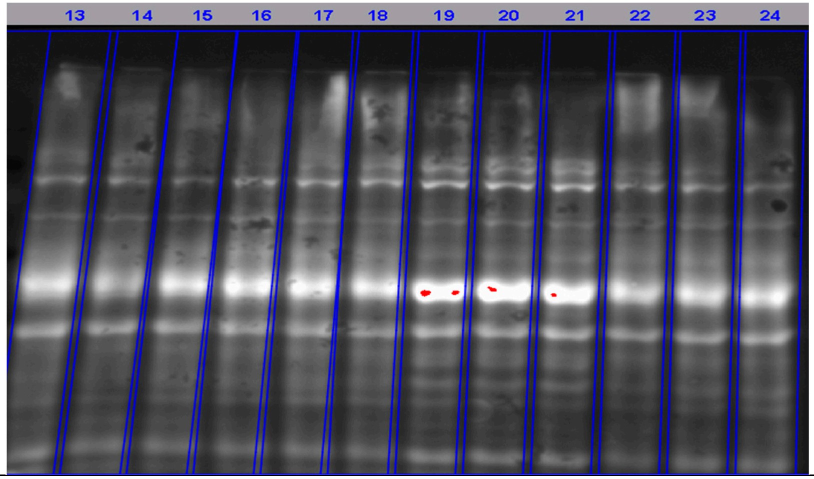

Cerebellum

Membrane 1 total protein	Membrane 2 Total protein
GAD-65 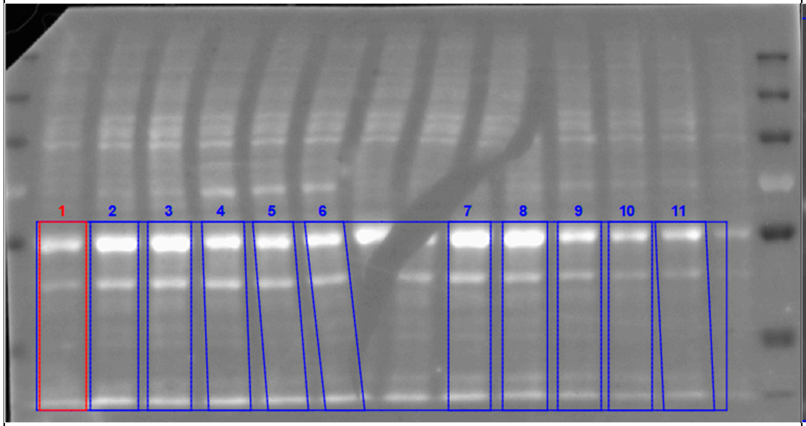	GAD-65 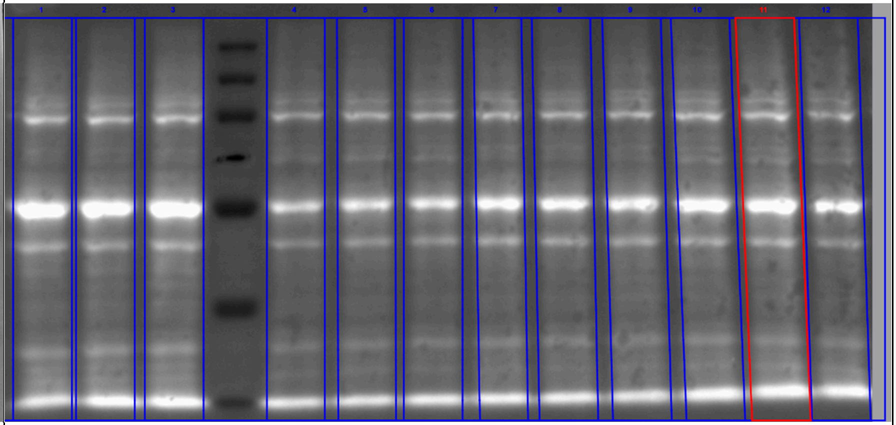
GAD-67 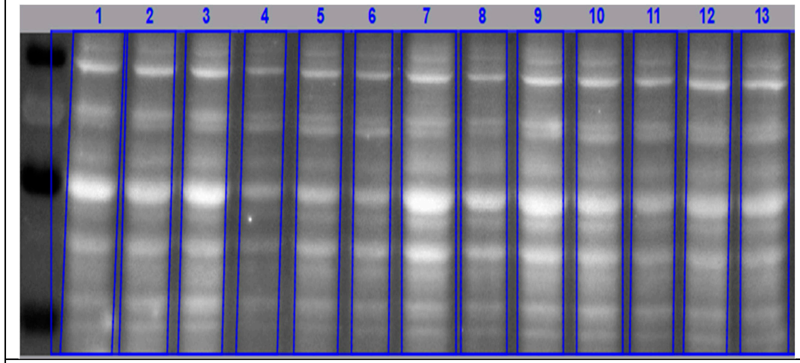	GAD-67 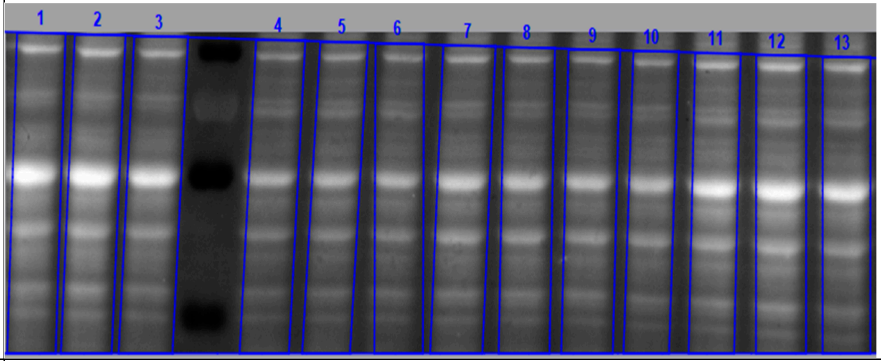

Membrane 1 total protein	Membrane 2 Total protein
PAG 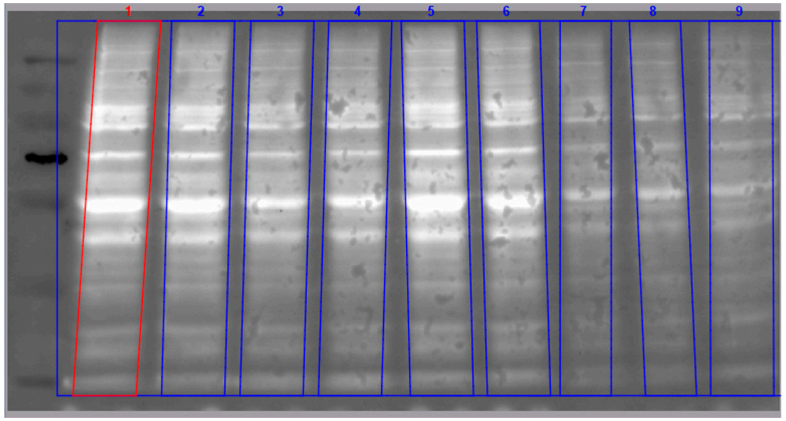	PAG 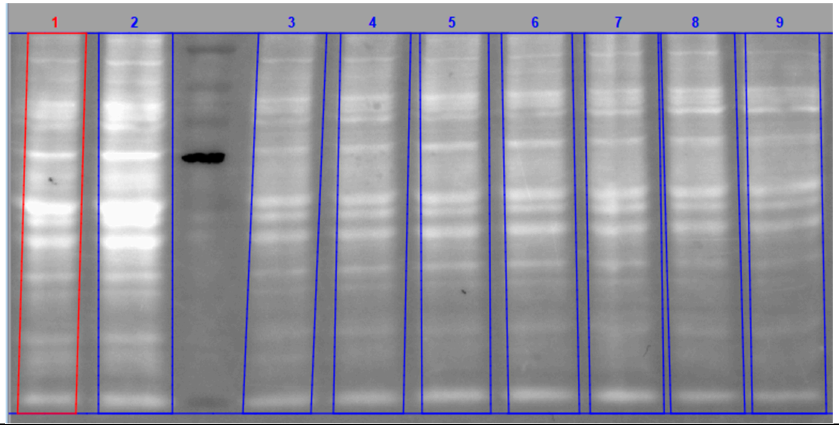
GLUD 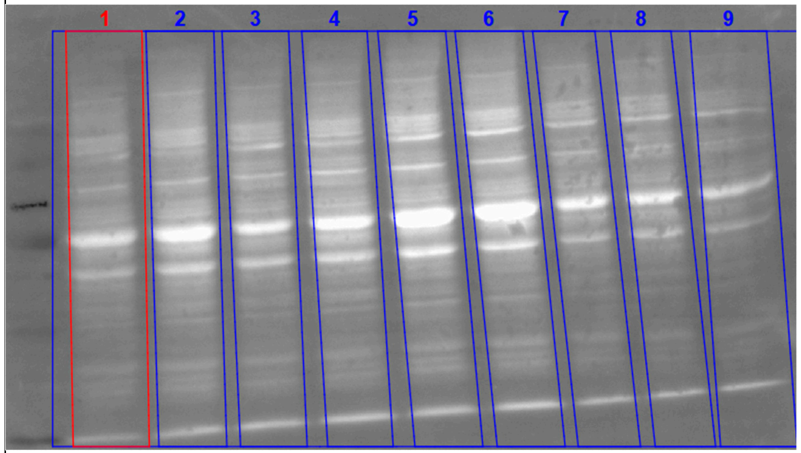	GLUD 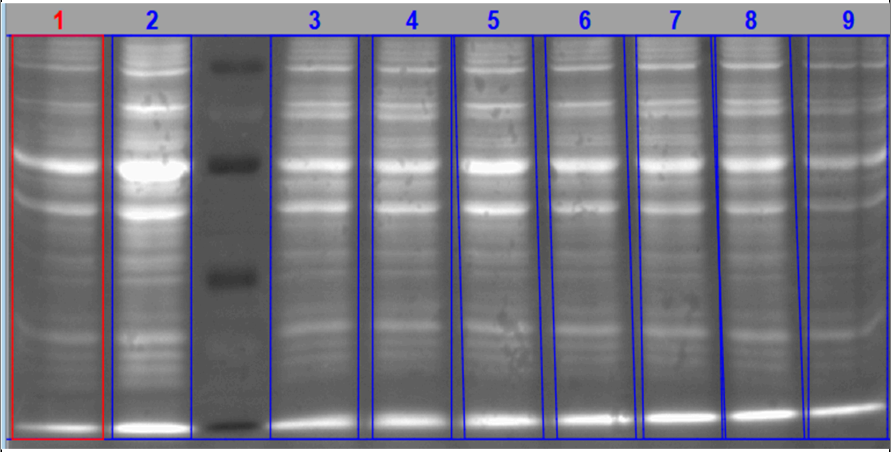

Membrane 1 total protein	Membrane 2 Total protein
VGAT<br>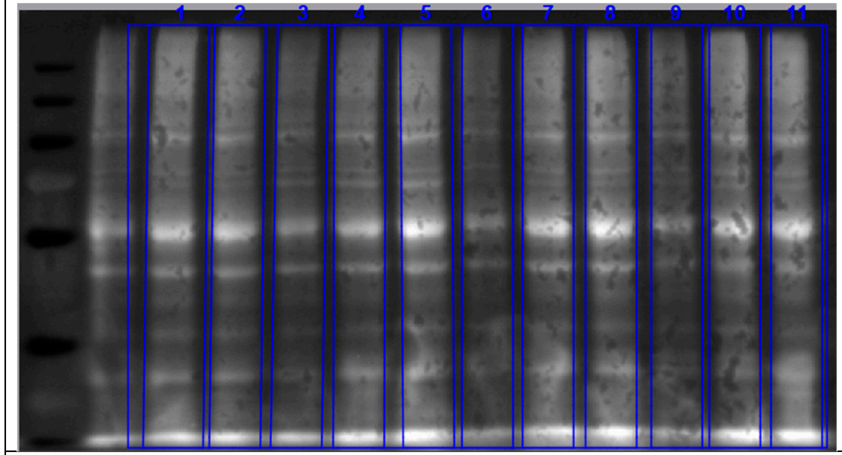 	VGAT<br>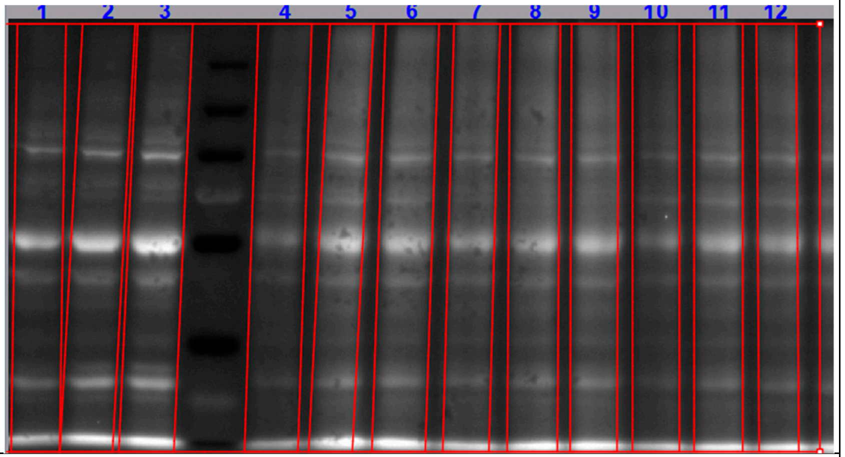
VGLUT<br>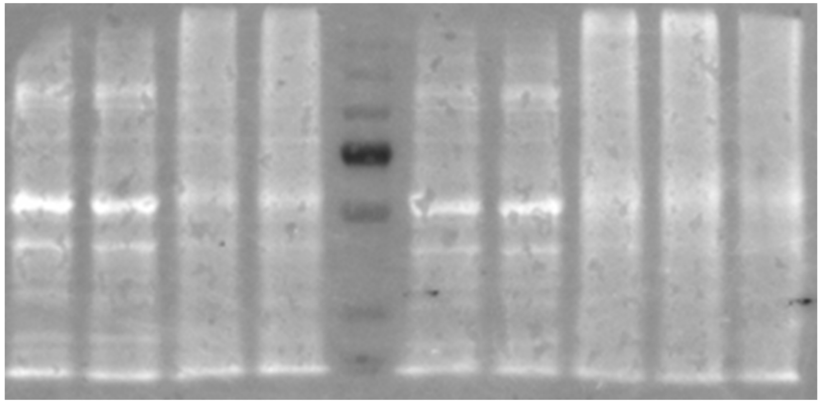	VGLUT<br>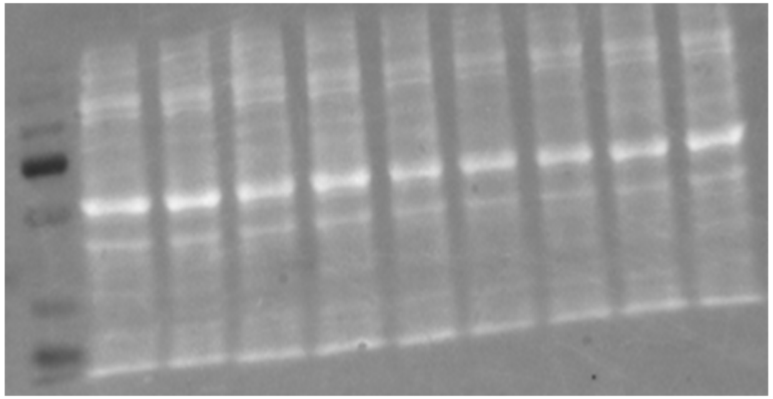
