# Supplementary material for: Alterations in the Components of the GABA–Glutamate System During ZIKV Infection: A Neuroscience Approach
Source: Int J Mol Sci. 2026 May 27;27(11):4833. doi: 10.3390/ijms27114833 (PMC13256588; doi:10.3390/ijms27114833)
Supplement: Supplementary file 1 [file ijms-27-04833-s001.zip › Supplement 4. Western Blot Fold Changes for Molecular Components of GABA and Glutamate Metabolism and Transport.pdf]

Supplement 4. Western Blot Fold Changes for Molecular Components of GABA and Glutamate Metabolism and Transport

Table S4.1 Cerebral cortex western blot assays

Brain area	Gen name	Fold change mock	Fold change infected	SD Mock-infected		P value
				Mock	Infected	
Cerebral cortex	GAD-65	0,92	1,41	0,3	0,48	0,007**
	GAD-67	1	0,91	0,13	0,16	0,15612**
	PAG	0,98	3.03	0,65	0,73	0,0004*
	GLUD1	1	0,75	0,24	0,19	0,0115**
	VGAT	1,08	1,5	0,55	0,97	0,244515**
	VGLUT 1	1	0,4	0,45	0,32	0,0184044**

Table S4.2 Cerebellum western blot assays

Brain area	Gen name	Fold change mock	Fold change infected	SD		P value
				Mock	Infected	
Cerebellum	GAD-65	1,08	1,19	0,55	1,22	0,558757*
	GAD-67	1	0,82	0,16	0,27	0,0552**
	PAG	1,07	2,25	0,323	0,55	0,00045 *
	GLUD1	1	0,88	0,41	0,34	0,5374**
	VGAT	1	0,86	0,43	0,28	0,3736**
	VGLUT1	1	1,37	0,5	0,83	,287386**

Note: The data obtained for mock and ZIKV groups for each marker were compared using the Wilcoxon-Mann-Whitney U test (*) and the Student's t-test (**) from the results obtained to determine the normality criteria. The data correspond to the analysis of four biological samples and three technical replicates.
