# Supplementary material for: Alterations in the Components of the GABA–Glutamate System During ZIKV Infection: A Neuroscience Approach
Source: Int J Mol Sci. 2026 May 27;27(11):4833. doi: 10.3390/ijms27114833 (PMC13256588; doi:10.3390/ijms27114833)
Supplement: Supplementary file 1 [file ijms-27-04833-s001.zip › Supplement 5. Cell Count Tables for GABA+ and Glutamate+ Neurons in Cerebral Cortex.pdf]

Supplement 5. Cell Count Tables for GABA+ and Glutamate+ Neurons in the Anterior and Posterior Cerebral Cortex

Table S5.1 GABA+ neuron distribution in the anterior cerebral cortex

Cerebral cortex layer / samples	Mock							Infected							
	1	2	3	4	5	Media	SD	1	2	3	4	5	Media	SD	Valor p
I	43	44	34	35	25	36	7,73	47	44	47	46	41	45	2,55	0,0397**
II	42	36	35	40	28	36	5,4	51	49	53	48	59	52	4,36	0.0079**
IIIa	45	30	20	30	26	30	9,23	40	41	46	40	40	41	261	0.0873
IIIb	35	23	23	24	22	25	5,41	45	39	33	43	38	40	4,67	0.0159**
V	129	74	77	75	66	84	25,39	150	160	128	132	110	136	19,54	0.0317**
VI	95	65	67	65	71	73	12,76	133	77	76	110	99	99	23,93	0.0317**
Totals	389	272	256	269	238	285	59,77	466	410	383	419	387	413	33,28	0.0317**

Table S5.2 GABA+ neuron distribution in the posterior cerebral cortex

Cerebral cortex layer / samples	Mock							Infected							
	1	2	3	4	5	Media	SD	1	2	3	4	5	Media	SD	Valor p
I	23	33	45	40	36	35	8,26	45	57	60	56	58	55	5,89	0,0159**
II	58	50	38	38	42	45	8,67	67	58	53	60	48	57	7,19	0.0635
III	41	28	25	28	29	30	6,22	46	46	42	49	41	45	3,27	0.0159**
IV	31	23	19	28	27	26	4,67	42	38	38	47	41	41	3,7	0.0079**
V	130	63	61	54	55	73	32,32	135	123	109	133	112	122	11,82	0.0556**
VI	72	23	63	41	37	47	19,95	60	76	73	71	90	74	10,79	0.0556**

**Totals**	355	220	251	229	226	256	56,46	395	398	375	416	390	395	14,79	0.0079**

Table S5.3. Glutamate+ neuron distribution in the anterior cerebral cortex

**Cerebral cortex layer / samples**	**Mock**							**Infected**							
	1	2	3	4	5	Media	SD	1	2	3	4	5	Media	SD	Valor p
**I**	78	141	119	96	43	95	37,7	70	65	40	64	37	55,2	15,45	0,0556
**II**	138	162	182	186	230	180	34,01	148	119	134	123	164	137,6	18,56	0,0556
**III**	147	160	176	166	195	169	18,02	137	144	127	121	181	142	23,54	0,0952
**IV**	149	159	179	169	216	174	25,8	123	129	119	116	200	137,4	35,33	0,0952
**V**	491	475	554	503	678	540	82,51	408	339	410	308	554	403,8	94,86	0,0635
**VI**	540	476	560	553	743	574	99,95	416	352	436	326	534	412,8	81,35	0,0159**
**Totals**	1543	1573	1770	1673	2105	1733	226,4	1302	1148	1266	1058	1670	1289	234,04	0,0317**

Table S5.4. Glutamate+ neuron distribution in the posterior cerebral cortex

**Cerebral cortex layer / samples**	**Mock**							**Infected**							
	1	2	3	4	5	Media	SD	1	2	3	4	5	Media	SD	Valor p
**I**	91	66	53	80	73	72,6	14,3	33	72	66	59	61	58,2	14,96	0,1746
**II**	195	204	198	221	207	205	10,1	166	121	146	146	126	141	18,03	0,0079**
**III**	182	156	178	210	209	187	22,8	144	123	150	145	118	136	14,44	0,0079**
**IV**	179	175	162	232	235	196,6	34,3	150	142	115	131	115	130,6	15,76	0,0079**
**V**	384	433	401	462	455	427	33,8	346	315	293	322	308	316,8	19,54	0,0079**
**VI**	426	394	415	536	527	459,6	66,7	324	372	300	256	330	316,4	42,58	0,0079**
**Totals**	1457	1428	1407	1741	1706	1548	162	1163	1145	1070	1059	1058	1099	50,83	0,0079**

Note: The data correspond to the counts of anterior and posterior cerebral cortex samples from five biological control (mock) replicates and five infected replicates. Three histological sections or technical replicates were selected in each case. Data were compared per layer using the Wilcoxon-Mann-Whitney U test.
