# Supplementary material for: Alterations in the Components of the GABA–Glutamate System During ZIKV Infection: A Neuroscience Approach
Source: Int J Mol Sci. 2026 May 27;27(11):4833. doi: 10.3390/ijms27114833 (PMC13256588; doi:10.3390/ijms27114833)
Supplement: Supplementary file 1 [file ijms-27-04833-s001.zip › Supplement 6. Counts and Densitometry of GABA+ and Glutamate+ in Cerebellum.pdf]

Supplement 6. Cell Count and Densitometry Tables for GABA+ and Glutamate+ Neurons in the Cerebellar Folia

Table S6.1. Number of GABA+ cells in th Purkinje cell layer (Mock)

Purkinje cell count GABA+ in mock group							
**Samples/Folias**	**I-II**	**III**	**IV-V**	**VI**	**IX**	**X**	**Media**
**1**	77	60	148	133	87	86	99
**2**	66	57	185	143	62	45	93
**3**	79	85	120	132	59	60	89
**4**	79	115	157	163	87	75	113
**5**	58	148	131	120	80	104	107
Media	72	93	148	138	75	74	100
**SD**	9,42	38,59	25,11	16,08	13,58	22,81	9,86

Table S6.2. Number of GABA+ cells in the Purkinje cell layer (Infected)

Purkinje cell count GABA+ in infected group							
**Samples/Folias**	**I-II**	**III**	**IV-V**	**VI**	**IX**	**X**	**Media**
**1**	45	47	39	60	37	54	47
**2**	28	42	50	19	46	52	40
**3**	39	51	33	46	32	46	41
**4**	42	66	82	83	16	44	56
**5**	20	71	54	42	49	39	46
Media	35	55	52	50	36	47	46
**SD**	10,47	12,5	18,96	23,61	13,1	6,08	6,36
p value	0,0079**	0,0952	0,0079**	0,0079**	0,0079**	0,0556	0,0079**

Table S6.3 GABA+ optical density in Purkinje cells of the mock group

Media value of the transmitted light scale (0-255) in mock group							
Samples/Folias	I-II	III	IV-V	VI	IX	X	Media
Mock1	131,259	129,626	109,23	96,491	93,811	103,554	110,662
Mock2	136,894	118,135	107,539	110,301	97,93	123,66	115,743
Mock3	138,331	132,375	100,62	106,567	91,241	118,477	114,602
Mock 4	118,765	128,324	110,764	118,113	101,759	110,79	114,753
Mock5	127,366	131,324	112,171	109,663	104,762	100,525	114,302
Media	130,523	127,957	108,065	108,227	97,901	111,401	114,012
DS	7,91	5,71	4,51	7,82	5,55	9,75	12,67

Table S6.4 GABA+ optical density in Purkinje cells of the infected group

Media value of transmited light scale (0-255) in infected group							
Samples/Folias	I-II	III	IV-V	VI	IX	X	Media
ZIKV2	76,793	76,983	80,925	59,119	76,958	79,506	75,047
ZIKV3	81,621	78,592	74,251	66,413	78,639	74,981	75,75
ZIKV4	74,336	79,441	71,64	68,172	64,787	60,614	69,832
ZIKV5	79,32	76,941	72,707	64,819	61,598	63,934	69,887
Media	86,831	84,48	75,446	67,893	71,987	71,234	76,312
p value	0,0159**	0,0079**	0,0079**	0,0079**	0,0079**	0,0079**	0,0022
DS	19,9	14,55	3,83	8,05	8,13	8,42	7,66

Table S2.5 Optical density of Glutamate+ cells in the granule cell layer of the mock group

Media value of transmited light scale (0-255) in mock group							
Samples/Folias	I-II	III	IV-V	VI	IX	X	Media
1	162,585	167,859	166,166	131,887	135,219	160,733	140,723
2	155,096	161,406	159,989	150,627	127,428	154,664	141,168
3	148,236	147,688	157,704	88,123	115,201	163,264	121,06
4	158,252	156,01	157,183	114,754	120,975	157,305	135,975
5	155,064	152,776	155,698	125,266	126,428	155,265	137,86
Media	155,846	157,148	159,348	122,131	125,05	158,246	135,357
SD	5,25	7,79	4,11	23,07	7,49	3,67	8,27

Table S2.6 Optical density of Glutamate+ cells in the granule cell layer of infected individuals

Media value of transmited light scale (0-255) in infected group							
Samples/Folias	I-II	III	IV-V	VI	IX	X	Media
1	161,179	151,697	152,517	173,328	179,323	180,767	168,505
2	169,335	167,206	172,075	163,174	184,373	174,639	168,695
3	151,662	145,616	156,024	153,443	122,413	162,189	150,116
4	141,554	159,06	167,596	158,201	175,766	184,274	162,756
5	150,321	145,663	150,433	142,052	191,873	196,835	159,006
Media	154,81	153,848	159,729	158,04	170,75	179,741	161,816
SD	10,69	8,46	9,57	11,59	27,69	12,73	7,71
p value	0,841	0,547	0,841	0,0159**	0,056**	0,0159**	0,0079**

Note: The data comprise counts and optical density for cerebellar folia samples from five biological control (mock) replicates and five infected replicates. Three histological sections or technical replicates were selected in each case. Data per cerebellum folia were compared using the Wilcoxon-Mann-Whitney U test.
