# Supplementary material for: Alterations in the Components of the GABA–Glutamate System During ZIKV Infection: A Neuroscience Approach
Source: Int J Mol Sci. 2026 May 27;27(11):4833. doi: 10.3390/ijms27114833 (PMC13256588; doi:10.3390/ijms27114833)
Supplement: Supplementary file 1 [file ijms-27-04833-s001.zip › Supplement 7. RIN measurements.pdf]

Supplement 7. Electropherograms of analyzed RNA samples

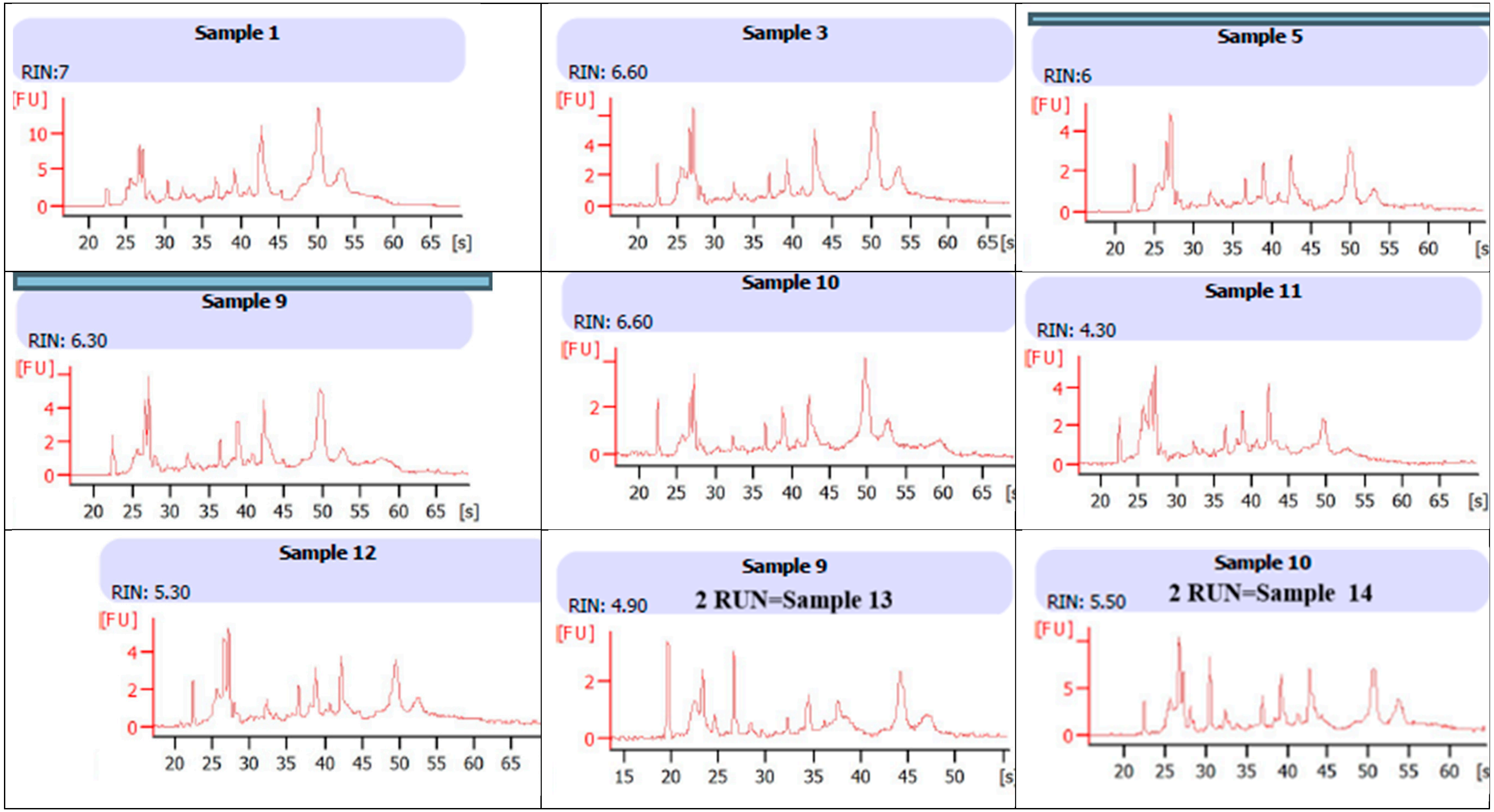

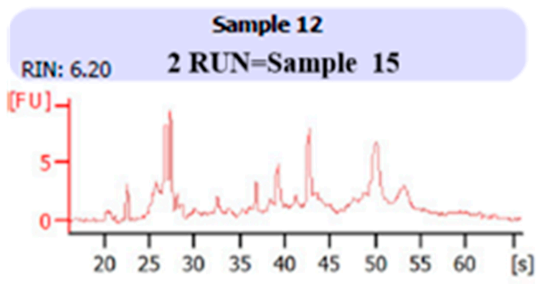

Summary of RIN values

Sample number	Treatment	RIN
1	Mock	7
3	Mock	6,6
8	Mock	6
9	Mock	6,3
10	Mock	6,6
11	ZIKV	4,3
12	ZIKV	5,3
13	ZIKV	4,9
14	ZIKV	5,5
15	ZIKV	6,2
